# ATG9A shapes the forming autophagosome through Arfaptin 2 and phosphatidylinositol 4-kinase IIIβ

**DOI:** 10.1083/jcb.201901115

**Published:** 2019-03-27

**Authors:** Delphine Judith, Harold B.J. Jefferies, Stefan Boeing, David Frith, Ambrosius P. Snijders, Sharon A. Tooze

**Affiliations:** 1 Molecular Cell Biology of Autophagy, The Francis Crick Institute, London, UK; 2 Bioinformatics and Biostatistics, The Francis Crick Institute, London, UK; 3 Proteomics, The Francis Crick Institute, London, UK

## Abstract

ATG9A is a multispanning membrane protein essential for autophagy. Normally resident in Golgi membranes and endosomes, during amino acid starvation, ATG9A traffics to sites of autophagosome formation. ATG9A is not incorporated into autophagosomes but is proposed to supply so-far-unidentified proteins and lipids to the autophagosome. To address this function of ATG9A, a quantitative analysis of ATG9A-positive compartments immunoisolated from amino acid–starved cells was performed. These ATG9A vesicles are depleted of Golgi proteins and enriched in BAR-domain containing proteins, Arfaptins, and phosphoinositide-metabolizing enzymes. Arfaptin2 regulates the starvation-dependent distribution of ATG9A vesicles, and these ATG9A vesicles deliver the PI4-kinase, PI4KIIIβ, to the autophagosome initiation site. PI4KIIIβ interacts with ATG9A and ATG13 to control PI4P production at the initiation membrane site and the autophagic response. PI4KIIIβ and PI4P likely function by recruiting the ULK1/2 initiation kinase complex subunit ATG13 to nascent autophagosomes.

## Abstract (precis)

ATG9A is essential during autophagosome biogenesis; however, the function of this multispanning membrane protein is not well defined. Judith et al. report that ATG9A vesicles deliver PI4KIIIβ to the autophagosome nucleation site to produce PI4P and initiate autophagosome formation.

## Graphical Abstract

**Figure:**
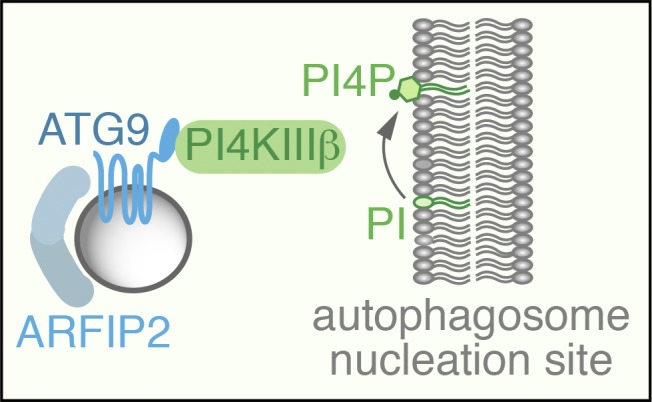

## Introduction

Macroautophagy, referred here as autophagy, is a dynamic, highly conserved, lysosomal-mediated degradative process necessary for eukaryote development, survival, and homeostasis. Autophagy can selectively eliminate damaged organelles, protein aggregates, and viral and bacterial pathogens. Autophagosome formation involves a cytoplasmic protein machinery acting on a membrane source to nucleate and form a phagophore, which will close to become a double-membrane autophagosome, which is then degraded after fusion with endolysosomes. The molecular machinery driving autophagy is composed of ATG proteins (Mizushima et al., 2011). While during nonselective and starvation-induced autophagy, autophagosomes originate from the ER, there may be multiple membrane sources contributing such as Golgi, recycling endosomes, and plasma membrane (Molino et al., 2017). Nucleation and expansion of the phagophore requires a flux of membrane lipids whose alteration could be deleterious for the outcome of the autophagy process (Dall’Armi et al., 2013).

ATG9 is the only multispanning ATG membrane protein essential for autophagy. In yeast, Atg9 vesicles are implicated in the delivery of membrane components to the initiation site or the preautophagosomal compartment (Reggiori et al., 2004; Yamamoto et al., 2012). Likewise, mammalian ATG9A (Young et al., 2006) is proposed to function in vesicular delivery to the initiation site or phagophore (Orsi et al., 2012; Karanasios et al., 2016).

Under nutrient-rich conditions, ATG9A is mainly located in the perinuclear region, colocalizing with medial and TGN markers of the Golgi complex, and partially with early and recycling endosomes (Young et al., 2006; Longatti et al., 2012; Orsi et al., 2012). During amino acid starvation, perinuclear ATG9A decreases concomitant with an increase in a vesicular population coincident with a partial colocalization with autophagosome markers (Orsi et al., 2012). Given its essential role, the partial colocalization of ATG9A with other ATG proteins during autophagy is surprising: it transiently interacts with the initiation site, also called the omegasome, and is not incorporated into a complete autophagosome (Orsi et al., 2012; Karanasios et al., 2016).

ATG9A vesicles are highly mobile, and their trafficking is controlled by nutrient-regulated signaling: in fed and starved cells, ATG9A trafficking from the Golgi complex is controlled by the ULK1/2 complex (Young et al., 2006; Chan et al., 2007). The ULK1/2 complex activation and recruitment to the omegasome is negatively regulated by mTORC1, the amino acid sensor and cell growth controller. ULK1/2 activates Myosin II to control ATG9A trafficking from the Golgi (Tang et al., 2011). ATG9A trafficking from the Golgi also requires BIF-1 (endophilin 1; Takahashi et al., 2011), working with Dynamin to drive the formation of ATG9A vesicles from recycling endosomes (Takahashi et al., 2016). The coat adaptors, AP-1 and AP-4, also mediate ATG9A trafficking and autophagy from the Golgi (Guo et al., 2012; Mattera et al., 2017). The interaction of ATG9A with AP-1 and AP-4 is regulated through phosphorylation by SRC kinase and ULK1 (Zhou et al., 2017). The role of lipids and enzymes that metabolize lipids in autophagy has been largely confined to understanding the class III phosphatidylinositol-3 kinase complex I and II (Burman and Ktistakis, 2010; Dall’Armi et al., 2013), but recent data suggest that sphingomyelin phosphodiesterase 1 controls ATG9A trafficking from the recycling endosome (Corcelle-Termeau et al., 2016). Other proteins contribute to ATG9A exit from recycling endosomes including p38IP, a p38 MAPK-interacting protein (Webber and Tooze, 2010), and Sorting nexin-18 (Søreng et al., 2018).

In amino acid starvation, ATG9A localizes to tubular-vesicular structures adjacent to nascent phagophores, called the ATG9 compartment (Orsi et al., 2012; Duke et al., 2014). Also found in nutrient starved yeast is the Atg9 reservoir (Mari et al., 2010) and highly mobile Atg9-positive vesicles (Yamamoto et al., 2012), supporting a conservation of ATG9 function in autophagy. While the exact role of ATG9A in mammals remains unknown, our hypothesis is that ATG9A acts in all stages of biogenesis and maturation of autophagosomes by delivering essential constituents and lipids.

The relationship of the ATG9 compartment and the highly mobile vesicles seen in both yeast and mammalian cells to the phagophore is not known. Although several properties of ATG9A are similar to those reported in yeast, in mammalian cells, ATG9A undergoes a complex trafficking pathway through the endocytic and secretory pathways (Young et al., 2006). Our work on the role of ULK1/2 and p38IP suggested that in amino acid starvation, the ATG9 compartment is derived from both the Golgi and recycling endosomes (Orsi et al., 2012). Rab1B, identified on ATG9A-GFP–positive vesicles (Kakuta et al., 2017), controls trafficking between the ER and Golgi (Wang et al., 2015b) and the Golgi and recycling endosomes (Marie et al., 2009). Ypt1 (Rab1 homologue) was previously found on yeast Atg9 vesicles (Kakuta et al., 2012; Yamamoto et al., 2012), and Rab1/Ypt1 mediate the TRAPPIII complex, which regulates ATG9A/Atg9 traffic (Kakuta et al., 2012; Lamb et al., 2016).

Using an antibody to endogenous ATG9A, we immunoisolated ATG9A-positive membranes and determined their composition by proteomics. We found Arfaptins (ARFIP1 and ARFIP2) to be components of ATG9A-positive membranes. ARFIPs are Bin/Amphiphysin/Rvs (BAR) domain–containing proteins, proposed to be required for sensing and generation of membrane curvature (Peter et al., 2004; Frost et al., 2009). The ARFIP BAR domain binds small GTPases such as ARFs, ARL1, and RAC1 (Lu et al., 2001; Shin and Exton, 2001; Tarricone et al., 2001; Man et al., 2011). ARFIPs contain an amphipathic helix (AH), which confers specificity for binding to phosphatidylinositol 4-phosphate (PI4P)–containing liposomes and the TGN (Cruz-Garcia et al., 2013). The binding of ARFIPs to the TGN requires both the AH and the BAR domain (Cruz-Garcia et al., 2013).

Despite their similar domain structure and subcellular localization, there are notable differences between ARFIP1 and 2. ARFIP1, shown to be required for secretory granule biogenesis (Gehart et al., 2012), is regulated by protein kinase D 1 (PKD1; Cruz-Garcia et al., 2013). ARFIP2 (also known as partner of Rac1, a Rho family GTPase [POR1]), in complex with ARF1, ARL1, and PKD2 regulates secretion of defined cargoes, matrix metalloproteases 2 and 7 (Eiseler et al., 2016). Moreover, ARFIP2 is recruited to high-curvature liposomes dependent on ARF1 (Ambroggio et al., 2013). We find that while depletion of ARFIP1 has no effect on autophagy, ARFIP2 positively regulates autophagy.

In addition, we show that the phosphatidylinositol 4 kinases, PI4KIIα and PI4KIIIβ, are components of ATG9A-positive membranes. As PI4KΙΙα acts at autophagosome–lysosome fusion (Wang et al., 2015a), we studied PI4KIIIβ and found it to be required for initiation of autophagosome formation. In line with this, we detected PI4P on early-stage autophagosomes, omegasomes, and phagophores. Our data reveal the importance of PI metabolism in the formation of the autophagosomes, and we propose that ARFIP2, by modulating the composition of ATG9A-positive membrane, controls amino acid starvation–induced autophagy.

## Results

### BAR domain–containing proteins are associated with ATG9A vesicles

**Figure 1.**
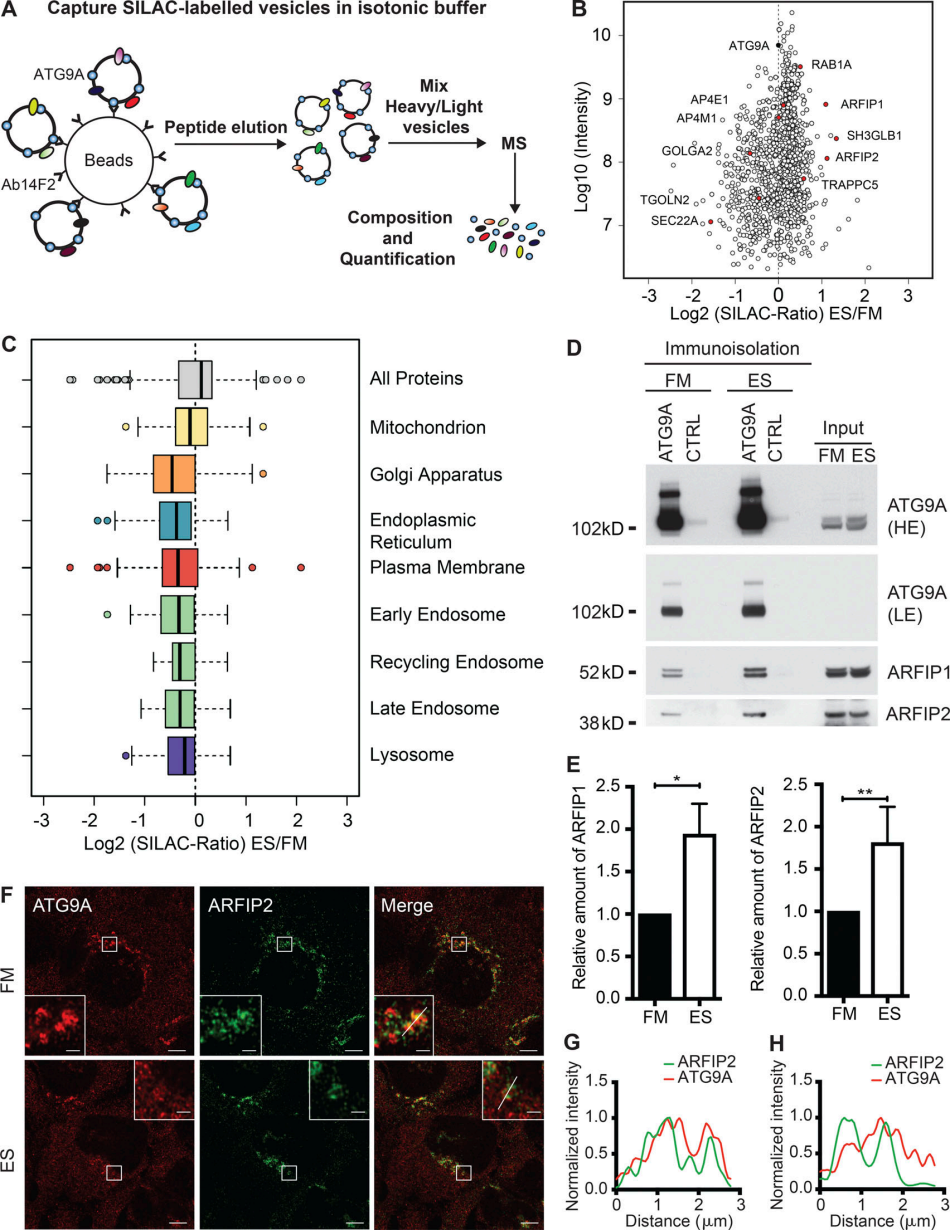
**Arfaptins are associated with ATG9A vesicles. (A)** Overview of the immunoisolation procedure used to purify ATG9A-positive membranes. Ab14F2 is a hamster monoclonal antibody raised against a C-terminal epitope (Webber and Tooze, 2010). **(B)** Scatterplot of proteins associated with ATG9A-positive membranes from cells incubated in FM or EBSS for amino acid depletion (ES). The x axis displays the Log2 of the SILAC ratio (ES/FM), and the y axis displays the Log10 of the intensity. ATG9A and examples of proteins enriched or depleted in ATG9A-positive membrane upon autophagy induction are highlighted. See Table S1. **(C)** Selected GO categories illustrate proteins associated with ATG9A-positive membranes from cells treated as in B. **(D)** HEK293A cells were incubated in FM or ES for 2 h, and the ATG9A-positive compartment was immunoisolated before detection of ATG9A, ARFIP1, and ARFIP2 by immunoblot. **(E)** Quantification of the relative amount of ARFIP1 and ARFIP1 after normalization to immunoisolated ATG9A in D. Statistical analysis using two-tailed unpaired Student’s *t* test, mean ± SEM, *n* = 3 experiments; *, P ≤ 0.05; **, P ≤ 0.01. **(F)** HEK293A cells were incubated in FM or ES for 2 h, fixed, and labeled using antibodies to ARFIP2 and ATG9A. Airyscan imaging. Scale bars, 5 µm; inset, 1 µm. Line scans of ARFIP2 and ATG9A labeling in FM (G) or ES (H) from F.

To identify the protein composition of ATG9A vesicles, nutrient-rich and amino acid–starved HEK293A cells were mechanically lysed, and ATG9A-positive membranes were immunoisolated using a hamster monoclonal antibody raised against human ATG9A or, as a control for specificity, hamster IgM (Fig. 1 A). Stable isotope labeling with amino acids in cell culture (SILAC) coupled with liquid chromatography/tandem mass spectrometry was used to compare the immunoisolated ATG9A-positive membranes from nutrient-rich and amino acid–starved cells. All proteins detected using a high stringency cutoff are shown in Fig. 1 B. Representative proteins in ATG9A-positive membranes enriched in amino acid starvation (Earle’s saline [ES]) are RAB1A, ARFIP1, ARFIP2, SH3GLB1 (BIF-1 or endophilin B), and TRAPPC5, or enriched in nutrient-rich medium (full medium [FM]) are GOLGA2, TGOLN2, and SEC22A (Fig. 1 B and Table S1). The levels of AP4 subunits, AP4E1 and AP4M2, are unchanged. We showed in FM that ATG9A is primarily in the Golgi apparatus, while in ES, ATG9A disperses to a peripheral location (Young et al., 2006). Validation of the ATG9A immunoisolation database on a gene ontology (GO) analysis shows the relative distribution of organelle-specific proteins (Fig. 1 C), confirmed that Golgi proteins (Golgi Apparatus GO category) are enriched in ATG9A-positive membranes isolated from FM and decreased in ES conditions (Fig. S1 A and Table S2).

BIF-1, ARFIP1, and ARFIP2 are BAR domain–containing proteins. Whereas BIF-1 is required for trafficking of ATG9A (Takahashi et al., 2011, 2016), little is known about the role of ARFIP1 and ARFIP2 in ATG9A trafficking or autophagy, and thus, we focused on the Arfaptins. In agreement with the SILAC data, ARFIP1 and ARFIP2 were detected on membranes isolated from cells in FM and enriched on ATG9A-positive membranes in ES (Fig. 1, D and E). By confocal microscopy, endogenous ARFIP1 and ARFIP2 were detected on immunoisolated mRFP-ATG9A–positive membranes bound to magnetic beads (Fig. S1 B).

Consistent with previous reports (Man et al., 2011), ARFIP1 and ARFIP2 are on the TGN (Fig. S1 C). To explore the biological relevance of ARFIP1 and ARFIP2 on ATG9A-positive membranes, we investigated whether these proteins have a role in autophagy. We depleted ARFIP1 using siRNA and, after incubation in ES, monitored the levels of the lipidated form of the ATG8 family member LC3B. LC3B is covalently lipidated (LC3B-II) during amino acid starvation on forming autophagosomes, is found on mature autophagosomes, and is widely used to monitor induction of autophagy. ARFIP1 depletion had no effect on LC3B lipidation or the formation of GFP-LC3B–positive puncta (Fig. S1, D–G). We also used WIPI2 puncta as a marker for earlier amino acid starvation events, as it binds PI3P at the phagophore, is required for LC3B lipidation, and remains associated with early autophagosomes but is not present on autolysosomes (Polson et al., 2010). ARFIP1 depletion had no effect on WIPI2 puncta formation (Fig. S1, F–H).

### ARFIP2 regulates starvation-induced autophagy and controls the trafficking of ATG9A

**Figure 2.**
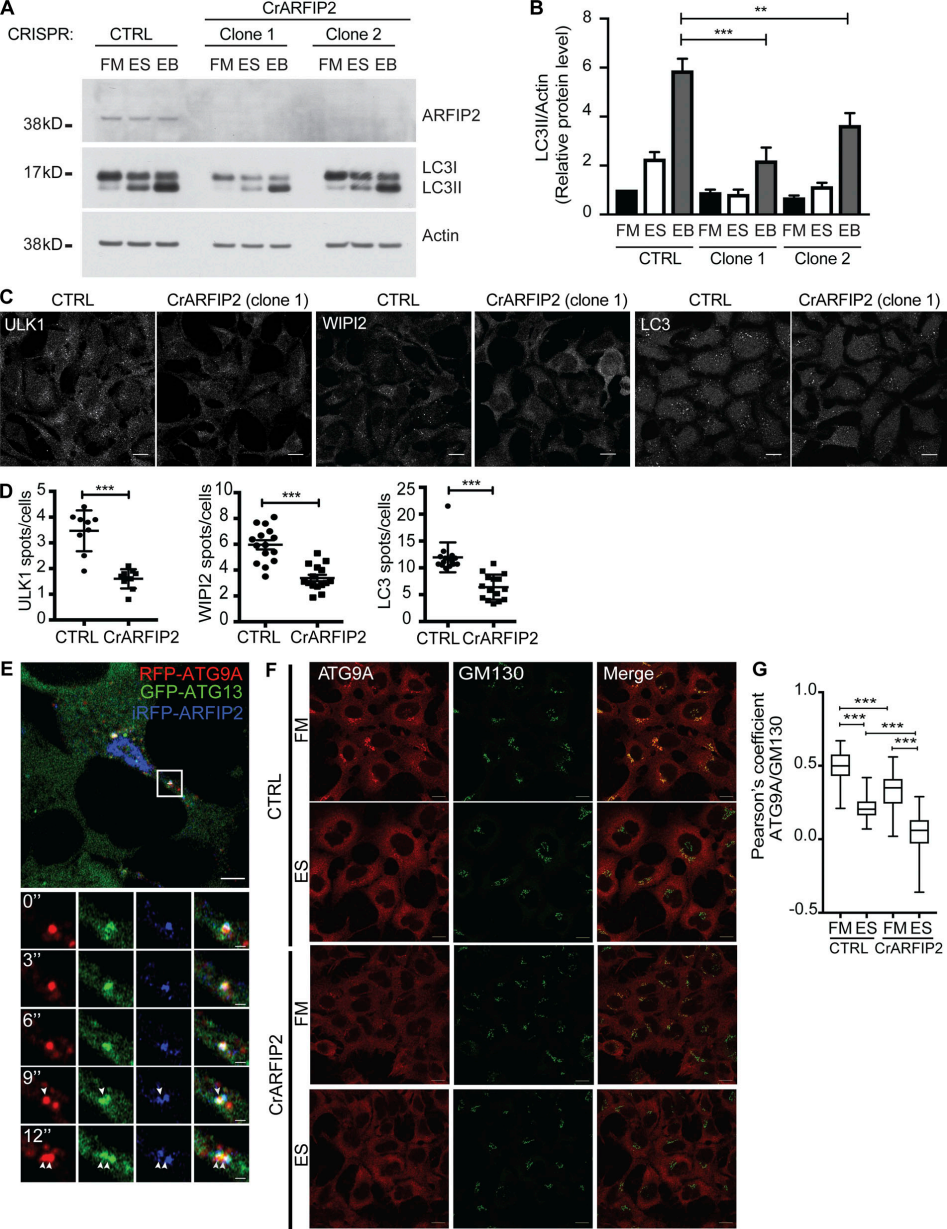
**ARFIP2 controls starvation-induced autophagy and the trafficking of ATG9A. (A)** HEK293A CTRL or ARFIP2 KO cells (CrARFIP2; clone 1 or clone 2) were incubated in FM or ES without or with Bafilomycin A1 (EB) for 2 h before immunoblotting for ARFIP2, Actin, and LC3B. **(B)** Quantification of A. Statistical analysis using one-way ANOVA with Tukey’s multiple comparisons test, mean ± SEM, *n* = 3 experiments; **, P ≤ 0.01; ***, P ≤ 0.001. **(C)** CTRL or CrARFIP2 KO (clone 1) cells were incubated in ES for 2 h, fixed, and labeled using antibodies to ULK1, WIPI2, or LC3B. Scale bars, 10 µm. **(D)** ULK1, WIPI2, and LC3B puncta in C were counted. Statistical analysis using two-tailed unpaired Student’s *t* test, mean ± SEM, *n* = 2 experiments for ULK1 puncta and *n* = 3 experiments for WIPI2 and LC3B puncta, 100 cells per independent experiment; ***, P ≤ 0.001. **(E)** HEK293A stably expressing mRFP-ATG9A and GFP-ATG13 were transfected with iRFP-ARFIP2, incubated in ES, and imaged live by Airyscan microscopy. Scale bars, 5 µm; inset, 1 µm. **(F)** CTRL or CrARFIP2 KO (clone 1) cells were incubated in FM or ES for 2 h before immunostaining for ATG9A and GM130. Scale bars, 10 µm. **(G)** Quantification of ATG9A and GM130 colocalization in F. Mean ± SEM, *n* = 3 experiments, Pearson’s coefficient was measured in 30 cells per condition per independent experiment, and statistical analysis was done using one-way ANOVA with Tukey’s multiple comparisons test; ***, P ≤ 0.001.

Next, we determined the effect of ARFIP2 depletion during autophagy. Analysis of the localization of ARFIP2 relative to ATG9A revealed an association in the perinuclear region in FM (Fig. 1, F and G). In ES, ATG9A showed the typical loss of its perinuclear localization and an increase in the dispersed peripheral localization, but there was only a slight change in the distribution of ARFIP2 (Fig. 1 F). However, a small population of ATG9A remained colocalized with ARFIP2 (Fig. 1 H). In siRNA depletion experiments performed in parallel with ARFIP1, ARFIP2 clearly reduced LC3B-II levels as well WIPI2 spot formation (Fig. S2, A–D). Therefore, we developed and characterized ARFIP2 CRISPR knockout (CrARFIP2 KO) cells. In CrARFIP2 KO clones 1 and 2, the loss of ARFIP2 decreased LC3B lipidation during amino acid starvation (Fig. 2, A and B). We determined the stage at which ARFIP2 functions by assessing the number of early (omegasomes and phagophores) and later autophagic structures in CrARFIP2 KO cells. Depletion of ARFIP2 upon starvation significantly reduced ULK1-positive, WIPI2-positive early structures, as well as the later LC3B-positive autophagosomes (Fig. 2, C and D; and Fig. S2, E and F).

Thus, as ARFIP2 is present on ATG9A vesicles, we asked if it behaved like ATG9A and is found associated with forming autophagosomes. We performed live-cell imaging of cells under amino acid starvation stably expressing GFP-ATG13 (a member of the ULK1/2 complex) and mRFP-ATG9A, and transiently expressed iRFP-ARFIP2 (Video 1 and Fig. 2 E). iRFP-ARFIP2 present on mRFP-ATG9 vesicles interacts with GFP-ATG13, which is recruited to omegasomes (Karanasios et al., 2016).

Given that ATG9A membranes contain ARFIP2, and that ARFIP2 is a positive regulator of autophagy, we assessed whether its loss affects the localization of ATG9A. In FM, loss of ARFIP2 caused a striking dispersion of ATG9A away from the juxtanuclear region compared with control (CTRL) cells (Fig. 2 F), quantified by the decreased overlap between ATG9A and GM130 (Fig. 2 G). In ES, ARFIP2 depletion increased the well-characterized dispersion of ATG9A from the Golgi (Fig. 2, F and G). These results suggest that ARFIP2 is required for autophagy and ATG9A trafficking in both fed and starved conditions.

**Figure 3.**
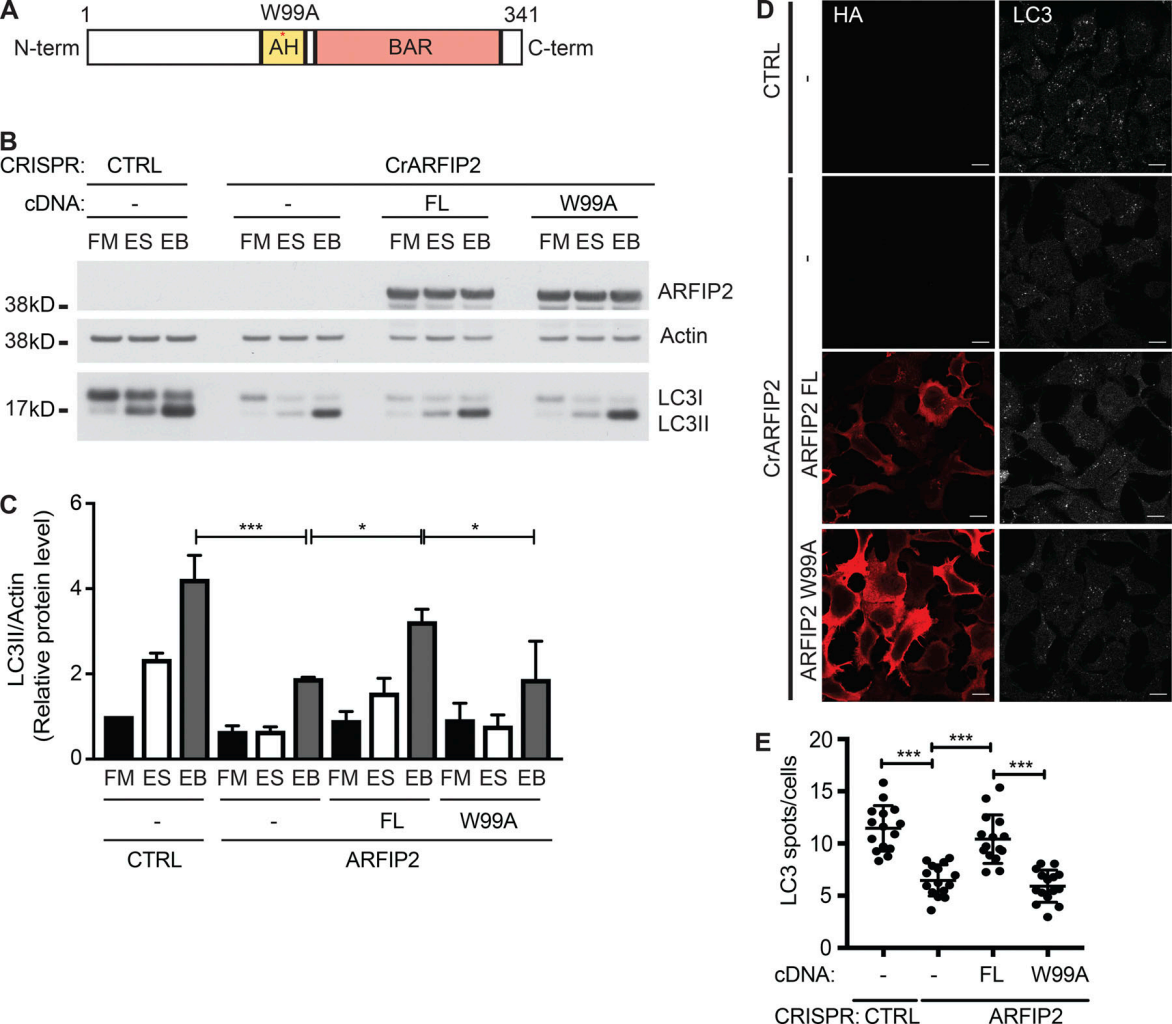
**The AH domain of ARFIP2 is required for autophagy. (A)** Schematic representation of human FL ARFIP2 showing the AH and BAR domain (BAR). An asterisk indicates the point mutation of phenylalanine to alanine in position 99 (W99A). **(B)** HEK293A CTRL or CrARFIP2 KO (clone 1) cells transfected with HA-ARFIP2 FL or HA-ARFIP2 W99A mutant were incubated in FM or ES without or with Bafilomycin A1 (EB) for 2 h before immunoblotting for HA, Actin, and LC3. **(C)** Quantification of B; statistical analysis using one-way ANOVA with Tukey’s multiple comparisons test, mean ± SEM, *n* = 8 experiments; *, P ≤ 0.05; ***, P ≤ 0.001. **(D)** CTRL or CrARFIP2 KO (clone 1) cells transfected with HA-ARFIP2 FL or HA-ARFIP2 W99A mutant were incubated in ES for 2 h, fixed, and labeled using antibodies to HA and LC3B. Scale bars, 10 µm. **(E)** LC3B puncta in CrARFIP2 KO (clone 1) cells expressing HA-ARFIP2 FL or ARFIP2 W99A were counted. Mean ± SEM, *n* = 3 experiments, 100 cells per independent experiment; ***, P ≤ 0.001.

ARFIP2 contains an AH adjacent to the BAR domain (Fig. 3 A). The AH is required for its Golgi localization and binding to PI4P, while the BAR domain senses or induces membrane curvature (Peter et al., 2004; Cruz-Garcia et al., 2013). Mutation of tryptophan 99 to alanine (W99A) in the AH abolishes the binding of ARFIP2 to PI4P-containing Golgi membranes (Cruz-Garcia et al., 2013). To validate the role of ARFIP2 in autophagosome formation and explore whether this depends on its ability to bind membranes upon starvation, we compared the rescue of LC3B lipidation and LC3B puncta in CrARFIP2 KO cells transfected with full-length (FL) wild-type (HA-ARFIP2 FL) to HA-ARFIP2 W99A (W99A). Rescue with FL modestly increases lipidation, while the W99A mutant does not (Fig. 3, B and C). Moreover, FL ARFIP2 rescued the number of LC3B-positive structures, while the W99A mutant did not (Fig. 3, D and E), indicating that the AH of ARFIP2 is required for its role in autophagosome formation.

### ATG9A modulates the PI4P pool as well as the PI4P kinases

**Figure 4.**
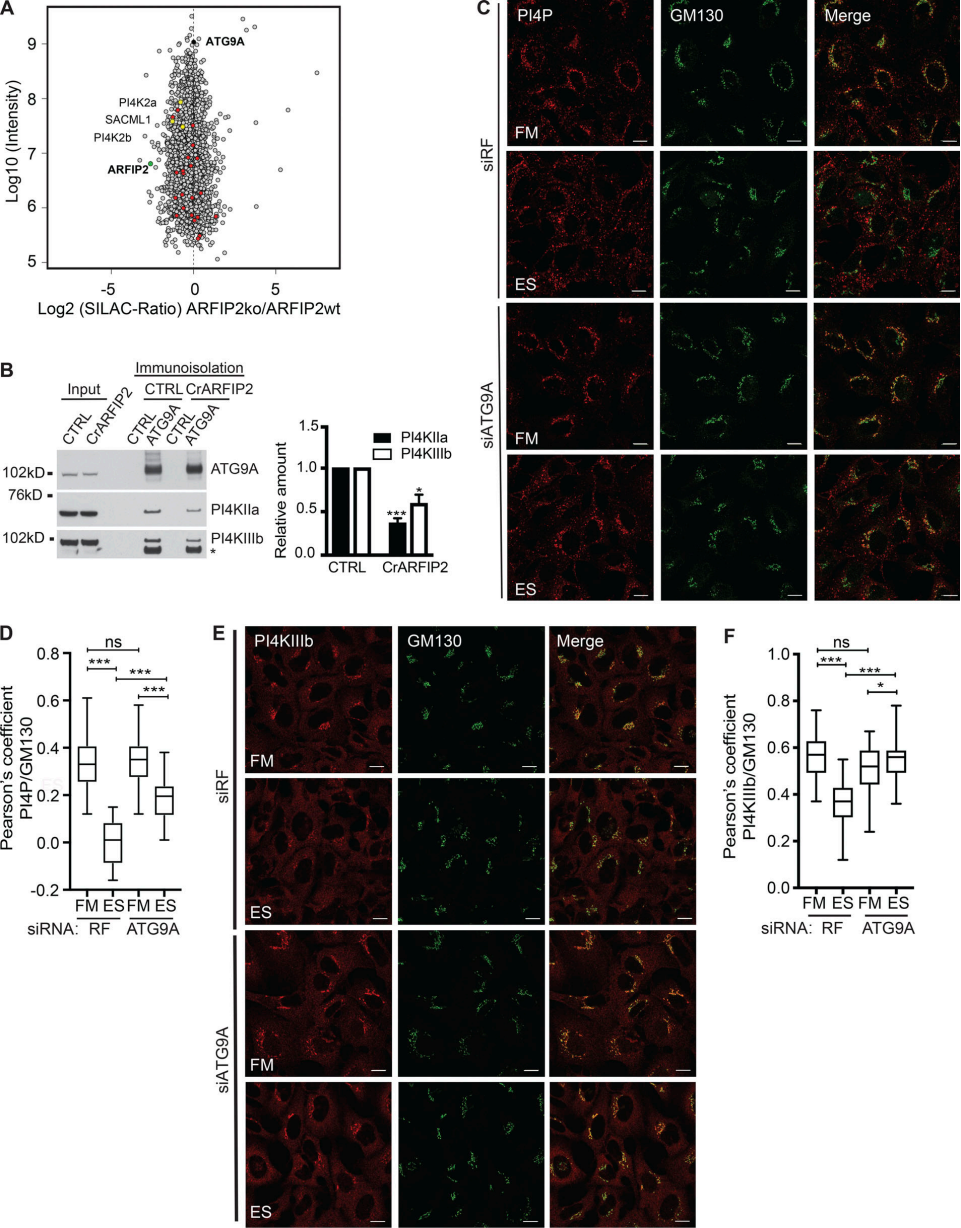
**ATG9A modulates the distribution of the cytoplasmic PI4P pool and PI4KIIIβ. (A)** Scatterplot of proteins associated with ATG9A-positive membranes from HEK293A CTRL or CrARFIP2 KO (clone 1) cells incubated in ES. The x axis displays the Log2 of the SILAC ratio (CrARFIP2 KO/CTRL), and the y axis displays the Log10 of the intensity. Highlighted in black and green are ATG9A and ARFIP2, respectively. Highlighted in red are proteins from the PI metabolic process GO category in ATG9A-positive membranes; yellow are proteins related to the production of PI4P depleted from ATG9A-positive membrane in CrARFIP2 KO (clone 1) cells. See Table S3. **(B)** CTRL or CrARFIP2 KO (clone 1) cells were incubated in ES for 2 h, and the ATG9A-positive compartment was immunoisolated before immunoblot for ATG9A, PI4KΙΙα, and PI4KIIIβ. Left: *, Nonspecific band. Quantification of the relative amount of PI4KIIIβ after normalization to immunoisolated ATG9A. Statistical analysis using two-tailed unpaired Student’s *t* test, mean ± SEM, *n* = 3 experiments; *, P ≤ 0.05; ***, P ≤ 0.001. **(C)** HEK293A cells were treated with RF siRNA or ATG9A siRNA for 72 h and then incubated in FM or ES for 2 h before immunostaining for PI4P and GM130. Scale bars, 10 µm. **(D)** Quantification of PI4P and GM130 colocalization in C. Mean ± SEM, *n* = 3 experiments, Pearson’s coefficient of 30 cells per condition per independent experiment was quantified, statistical analysis using one-way ANOVA with Tukey’s multiple comparisons test; ***, P ≤ 0.001. **(E)** HEK293A cells were treated with RF or ATG9A siRNA for 72 h and then incubated in FM or ES for 2 h before immunostaining for PI4KIIIβ and GM130. Scale bars, 10 µm. **(F)** Quantification of PI4KIIIβ and GM130 colocalization in E. Mean ± SEM, *n* = 3 experiments, Pearson’s coefficient of 30 cells per condition per independent experiment was quantified, statistical analysis using one-way ANOVA with Tukey’s multiple comparisons test; *, P ≤ 0.05; ***, P ≤ 0.001.

As our data suggest that ARFIP2 modulates ATG9A trafficking, we assessed whether depletion of ARFIP2 would modify the composition of the ATG9A vesicles upon amino acid starvation. Using immunoisolation-SILAC coupled with liquid chromatography/tandem mass spectrometry, we compared immunoisolated ATG9A-positive membranes from CTRL and CrARFIP2 KO cells in ES (Fig. 4 A). Among the proteins depleted in ATG9A-positive membranes immunoisolated from CrARFIP2 KO cells (Table S3) were several proteins involved in PI4P metabolism (Fig. 4 A, yellow dots).

These data and the fact that ARFIP2 localized at the Golgi apparatus led us to investigate the two major PI4-kinases present on the Golgi apparatus: PI4KIIα and PI4KIIIβ (Graham and Burd, 2011; Balla, 2013). We confirmed that both PI4KIIα and PI4KIIIβ are present on ATG9A membranes in FM and ES conditions (Fig. S3 A). In CrARFIP2 KO cells, both proteins were decreased under starvation conditions in ATG9A-immunoisolated membranes (Fig. 4 B), suggesting that ARFIP2 controls the localization of PI4-kinases on ATG9A-positive membranes. Immunoprecipitation experiments using detergent-solubilized cells revealed that ATG9A interacts with both PI4KIIα and PI4KIIIβ (Fig. S3 B).

As ATG9A-positive membranes contain PI4KIIα and PI4KIIIβ, we assessed whether ATG9A-positive membranes contain PI4P. We first compared the localization of PI4P to ATG9A using an antibody that recognizes PI4P (Hammond et al., 2009). In FM, PI4P is concentrated at the perinuclear region of the cells, localizing with GM130 (Fig. 4, C and D). Interestingly, in ES, the PI4P pool at the Golgi disperses, with a decrease in the overlap between PI4P and GM130 (Fig. 4, C and D). In both fed and starved cells, PI4P labeling showed PI4P-positive puncta localized with ATG9A (Fig. S3, C–E). To understand the dynamics of this punctate vesicle population, live-cell imaging was done using a validated PI4P biosensor, GFP-P4MX2, to follow the PI4P on ATG9A vesicles in starved cells. P4MX2 contains the SidM domain of the secreted effector protein SidM from the bacterial pathogen *Legionella pneumophila*, which labels PI4P (Del Campo et al., 2014; Hammond et al., 2014). Imaging mRFP-ATG9A and GFP-P4MX2 revealed that ATG9A-positive vesicles transiting under amino acid starvation contain PI4P (Video 2 and Fig. S3 F).

We tested if ATG9A is required for the relocalization or maintenance of the PI4P pool upon starvation. In FM, ATG9A depletion does not affect the colocalization of PI4P with GM130, while in ES, in ATG9A-depleted cells, more PI4P remains in the Golgi region associated with GM130 (Fig. 4, C and D) compared with RISC-free (RF) siRNA. These results imply that upon amino acid starvation, ATG9A facilitates the increase in PI4P in peripheral membrane compartments.

We focused on PI4KIIIβ, as PI4KIIα has already been shown to play a role in autophagosome maturation (Wang et al., 2015a), and investigated the relationship between PI4KIIIβ and ATG9A. Under FM conditions, PI4KIIIβ is concentrated at the perinuclear region of the cells in association with ATG9A, and upon ES treatment, PI4KIIIβ remained localized with a fraction of the dispersed ATG9A (Fig. S3 G). We assessed the effect of ATG9A depletion on the localization of PI4KIIIβ. In FM, PI4KIIIβ is concentrated at the Golgi in association with GM130, in both RF and ATG9A-depleted cells (Fig. 4, E and F). Upon ES starvation in RF, the overlap between PI4KIIIβ and GM130 decreased (implying a redistribution of PI4KIIIβ to peripheral membranes), while strikingly, in ATG9A depleted cells, PI4KIIIβ remained at the Golgi, colocalizing with GM130 (Fig. 4, E and F).

### ARFIP2 modulates the distribution of PI4P and PI4KIIIβ

**Figure 5.**
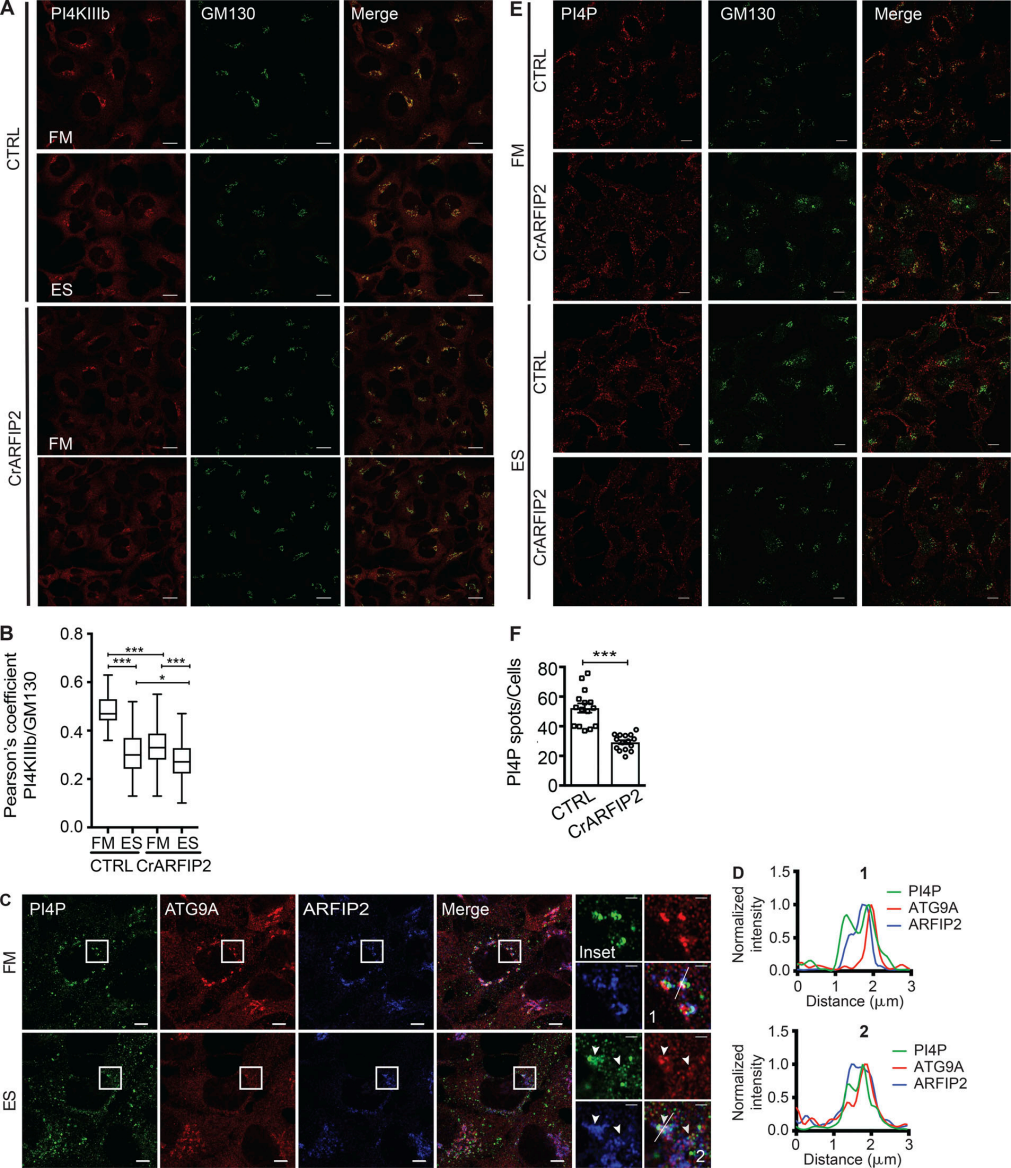
**ARFIP2 controls the cytoplasmic PI4P pool and PI4KIIIβ localization. (A)** HEK293A CTRL or CrARFIP2 KO (clone 1) cells incubated in FM or ES for 2 h before immunostaining for PI4KIIIβ and GM130. Scale bars, 10 µm. **(B)** Quantification of PI4KIIIβ and GM130 colocalization in A. Mean ± SEM, *n* = 3 experiments, Pearson’s coefficient was measured in 30 cells per condition per independent experiment, statistical analysis using one-way ANOVA with Tukey’s multiple comparisons test; *, P ≤ 0.05; ***, P ≤ 0.001. **(C)** HEK293A cells were incubated in FM or ES for 2 h, fixed, and labeled using antibodies to PI4P, ATG9A, and ARFIP2. Airyscan imaging. Scale bars, 5 µm; inset, 1 µm. Arrowheads indicate colocalized structures. **(D)** Line scans of C in FM (1) and ES (2). **(E)** CTRL or CrARFIP2 KO (clone 1) cells were incubated in FM or ES for 2 h before immunostaining for PI4P and GM130. Scale bars, 10 µm. **(F)** PI4P puncta in E were counted. Statistical analysis using two-tailed unpaired Student’s *t* test, mean ± SEM, *n* = 3 experiments, 100 cells per condition per independent experiment were quantified; ***, P ≤ 0.001.

Since ARFIP2 is on ATG9A vesicles (Fig. 1) and regulates the trafficking of ATG9A (Fig. 2, F and G), we determined whether ARFIP2 is involved in PI4KIIIβ distribution and examined the location of the PI4KIIIβ in CrARFIP2 KO cells. In FM, loss of ARFIP2 caused a dispersion of PI4KIIIβ into the peripheral regions as shown by the decreased overlap between PI4KIIIβ and GM130 (Fig. 5, A and B). This was similar to the effect on ATG9A localization after loss of ARFIP2 (Fig. 2, F and G). In ES, in both CTRL and CrARFIP2 KO cells, we observed a decreased colocalization between PI4KIIIβ and GM130 compared with the FM CTRL (Fig. 5, A and B). The dispersion of PI4KIIIβ in amino acid–starved cells significantly increased in CrARFIP2 KO cells compared with CTRL starved cells (Fig. 5 B).

To understand the functional relationship between ARFIP2 and PI4P, we assessed whether PI4P localized with ARFIP2 on ATG9A-positive membranes. In FM and ES condition, PI4P is found on ATG9A and ARFIP2-positive membranes (Fig. 5, C and D). We asked whether ARFIP2 is required for the peripheral redistribution of PI4P puncta observed upon starvation. In FM, in CrARFIP2 KO cells, the Golgi localization of PI4P was reduced (Fig. 5 E) compared with CTRL cells, where PI4P is concentrated at the Golgi. In ES conditions, in both CTRL and CrARFIP2 KO cells, PI4P no longer colocalizes with the GM130-positive Golgi membranes (Fig. 5 E). Moreover, PI4P-positive puncta were reduced in starved CrARFIP2 KO cells (Fig. 5 F). These data suggest that ARFIP2 regulates autophagosome formation, likely by modulating ATG9A trafficking, and subsequently PI4P localization and PI4KIIIβ distribution.

### PI4P and PI4KIIIβ are on autophagosomal structures

**Figure 6.**
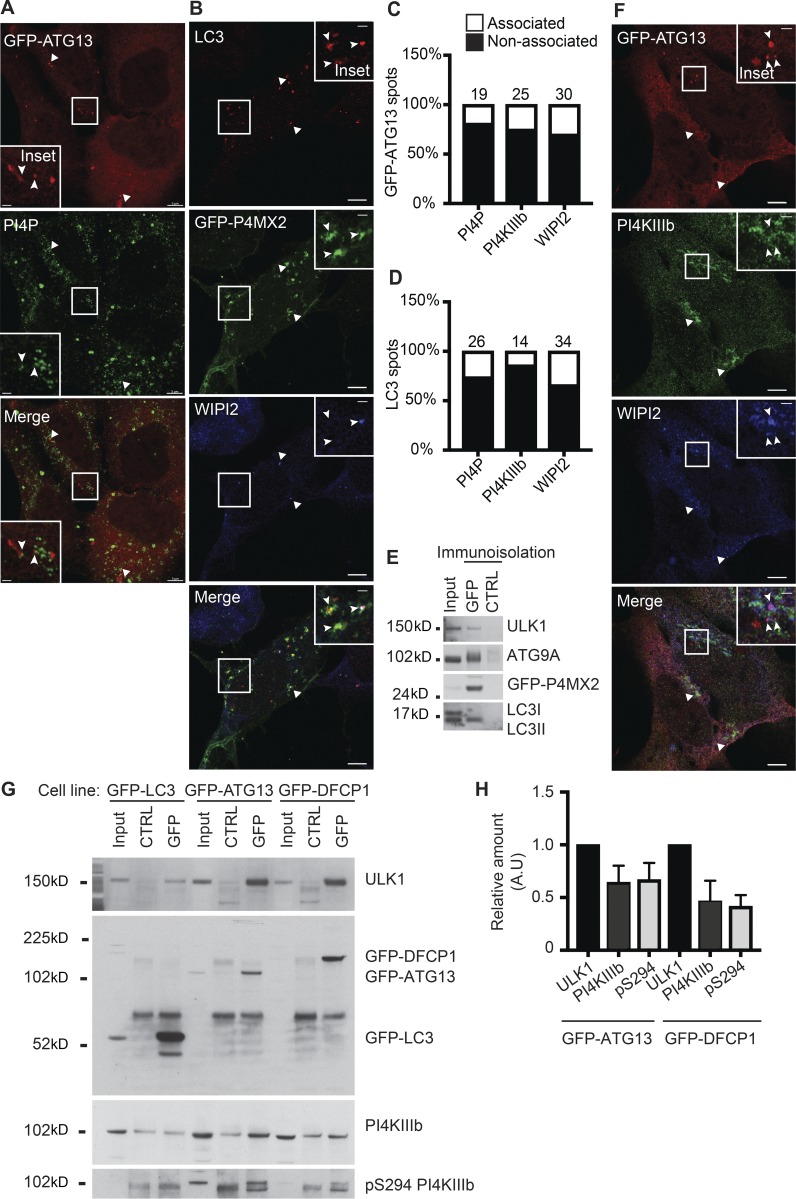
**PI4P and PI4KIIIβ are associated with autophagosomal structures. (A)** HEK293A cells stably expressing GFP-ATG13 were incubated in ES for 2 h, fixed, and labeled using antibodies to PI4P. Airyscan imaging. Scale bars, 5 µm; inset, 1 µm. **(B)** HEK293A cells transfected with GFP-P4MX2 were incubated in ES for 2 h, fixed, and labeled using antibodies to LC3B and WIPI2. Airyscan imaging. Scale bars, 5 µm; inset, 1 µm. **(C and D)** Quantification of the percentage of GFP-ATG13 (C) or LC3 (D) spots associating with PI4P, PI4KIIIβ, and WIPI2 (analysis of 100 spots). **(E)** HEK293A cells transfected with GFP-P4MX2 were incubated in ES for 2 h, and the GFP-P4MX2-positive compartment was immunoisolated before immunoblot for GFP, ULK1, ATG9A, and LC3B. **(F)** HEK293A-GFP-ATG13 cells were incubated in ES for 2 h, fixed, and labeled using antibodies to PI4KIIIβ and WIPI2. Airyscan imaging. Scale bars, 5 µm; inset, 1 µm. In A, B, and F, arrowheads indicate colocalized structures. **(G)** HEK293A cells stably expressing GFP-LC3B, GFP-ATG13, or GFP-DFCP1 were incubated in ES for 2 h, and the GFP-positive compartment was immunoisolated before immunoblot for GFP, ULK1, PI4KIIIβ, and pS294 PI4KIIIβ. **(H)** Quantification of the relative amount of ULK1, PI4KIIIβ, and pS294 PI4KIIIβ in GFP-ATG13- and GFP-DFCP1–positive compartment. Mean ± SEM, *n* = 3 experiments.

We then aimed to identify the stage at which autophagic membranes acquire PI4P and PI4KIIIβ. It has been shown that autophagosomes contain PI4P (Wang et al., 2015a); however, nothing is known about the earlier structures, the omegasome and phagophore. To test if these earlier membranes contain PI4P, we investigated the localization of PI4P relative to ATG13-, DFCP1-, and LC3B-positive structures. Anti-PI4P staining showed that GFP-ATG13–positive (Fig. 6 A) and DFCP1-positive (Fig. S4 A) structures are found in close proximity to PI4P-positive membranes. We found that PI4P colocalizes with, and encircles, GFP-LC3B–positive structures (Fig. S4 B). GFP-P4MX2 labeling confirmed that PI4P was present on WIPI2- and LC3B-positive phagophores and autophagosomes (Fig. 6 B). In relative terms, ∼20% of GFP-ATG13–positive membranes are associated with PI4P compared with ∼30% of WIPI2. Approximately 25% of LC3-positive membranes are associated with PI4P compared with ∼35% of WIPI2 (Fig. 6, C and D). To validate the PI4P localization to autophagic structures, we immunoisolated GFP-P4MX2–positive membranes from starved cells and observed that the immunoisolated membranes contained ATG9A, ULK1, and LC3B-II (Fig. 6 E), in support of the conclusion that PI4P is present on both ATG9A vesicles and the forming autophagosomes.

We examined the localization of PI4KIIIβ on early autophagy structures and could detect PI4KIIIβ on GFP-ATG13– and WIPI2-positive membranes (Fig. 6 F). Again, in relative terms, ∼25% of GFP-ATG13–positive membranes are associated with PI4KIIIβ, while only ∼15% of LC3-positive membranes are associated with PI4KIIIβ (Fig. 6, C and D). Finally, in a comparative analysis, early autophagy membranes were immunoisolated and analyzed in the same Western blot to compare the levels of PI4KIIIβ on each (Fig. 6 G). Immunoisolated GFP-ATG13– and GFP-DFCP1–positive membranes, which contain ULK1 and GABARAP (another member of the ATG8 family), also contain PI4KIIIβ and its phosphorylated activated form (Hausser et al., 2005; Figs. 6 G and S4 C), while the GFP-LC3B–positive membranes have very low levels of ULK and undetectable PI4KIIIβ. By comparison, GFP-ATG13 and GFP-DFCP1 membranes (after normalization to ULK1 levels) contained PI4KIIIβ and activated PI4KIIIβ pSer294, and both were slightly more enriched in GFP-ATG13–positive membranes (Fig. 6 H).

### PI4KIIIβ regulates PI4P and ATG9A trafficking

**Figure 7.**
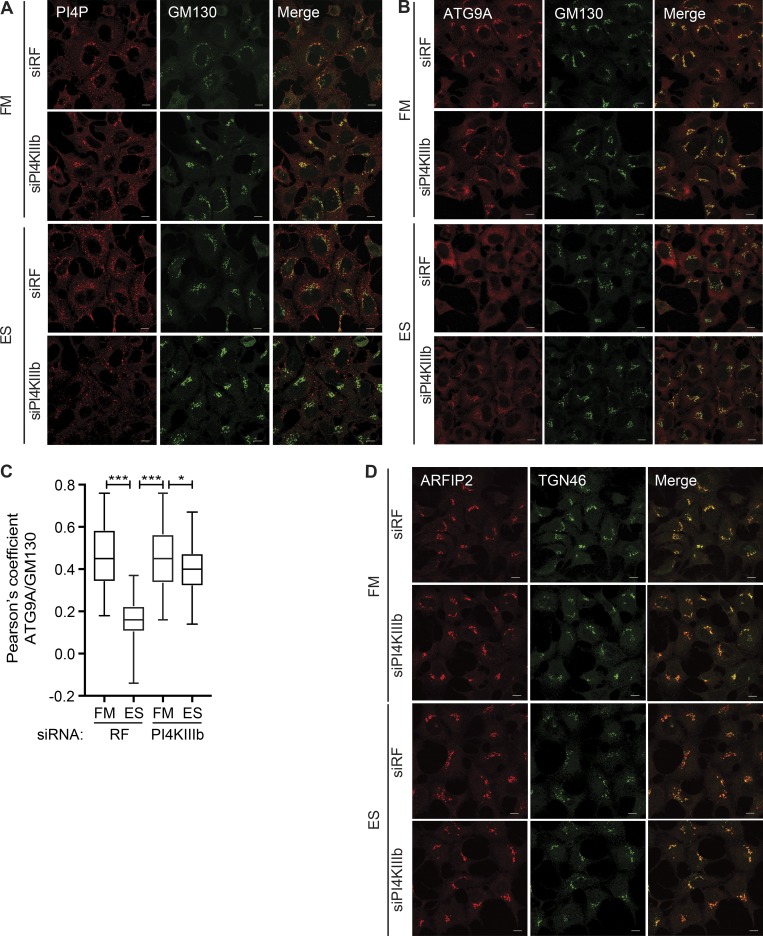
**PI4KIIIβ regulates PI4P and ATG9A trafficking. (A)** HEK293A cells were treated with RF or PI4KIIIβ siRNA for 72 h, incubated in FM or ES for 2 h, and immunostained for PI4P and GM130. Scale bars, 10 µm. **(B)** HEK293A cells were treated with RF or PI4KIIIβ siRNA for 72 h and then incubated in FM or ES for 2 h, fixed, and labeled using antibody to ATG9A and GM130. Scale bars, 10 µm. **(C)** Quantification of ATG9A and GM130 colocalization in B; statistical analysis using one-way ANOVA with Tukey’s multiple comparisons test, mean ± SEM, *n* = 3 experiments, Pearson’s coefficient of 30 cells per condition per independent experiment were quantified; *, P ≤ 0.05; ***, P ≤ 0.0.001. **(D)** HEK293A cells were treated with RF or PI4KIIIβ siRNA for 72 h, incubated in FM or ES for 2 h, fixed, and labeled using antibodies to ARFIP2 and TGN46. Scale bars, 10 µm.

As PI4KIIIβ occurs on early autophagic membranes (Fig. 6), we examined the role of PI4KIIIβ in the redistribution of PI4P upon induction of autophagy seen in Fig. 5 E. Upon depletion of PI4KIIIβ in amino acid starvation, PI4P puncta decrease, suggesting that PI4KIIIβ is responsible for the redistribution of the peripheral PI4P-positive puncta (Fig. 7 A). These data imply that PI4KIIIβ controls production and relocalization of peripheral PI4P puncta upon amino acid starvation.

We examined whether PI4KIIIβ was required for ATG9A trafficking. In FM, PI4KIIIβ siRNA depletion had no effect on ATG9A resident in the perinuclear region of the cell colocalizing with GM130 (Fig. 7, B and C). However, in ES, the translocation of ATG9A to the peripheral puncta was significantly inhibited by PI4KIIIβ depletion (Fig. 7, B and C). This indicates that the peripheral redistribution of ATG9A upon starvation requires PI4KIIIβ, and that PI4KIIIβ plays a key role in starvation-induced ATG9A trafficking. We tested whether PI4KIIIβ was required for ARFIP2 localization and found that PI4KIIIβ depletion did not affect ARFIP2 localization in either FM or ES conditions (Fig. 7 D).

### PI4KIIIβ regulates autophagy at the earliest stage

**Figure 8.**
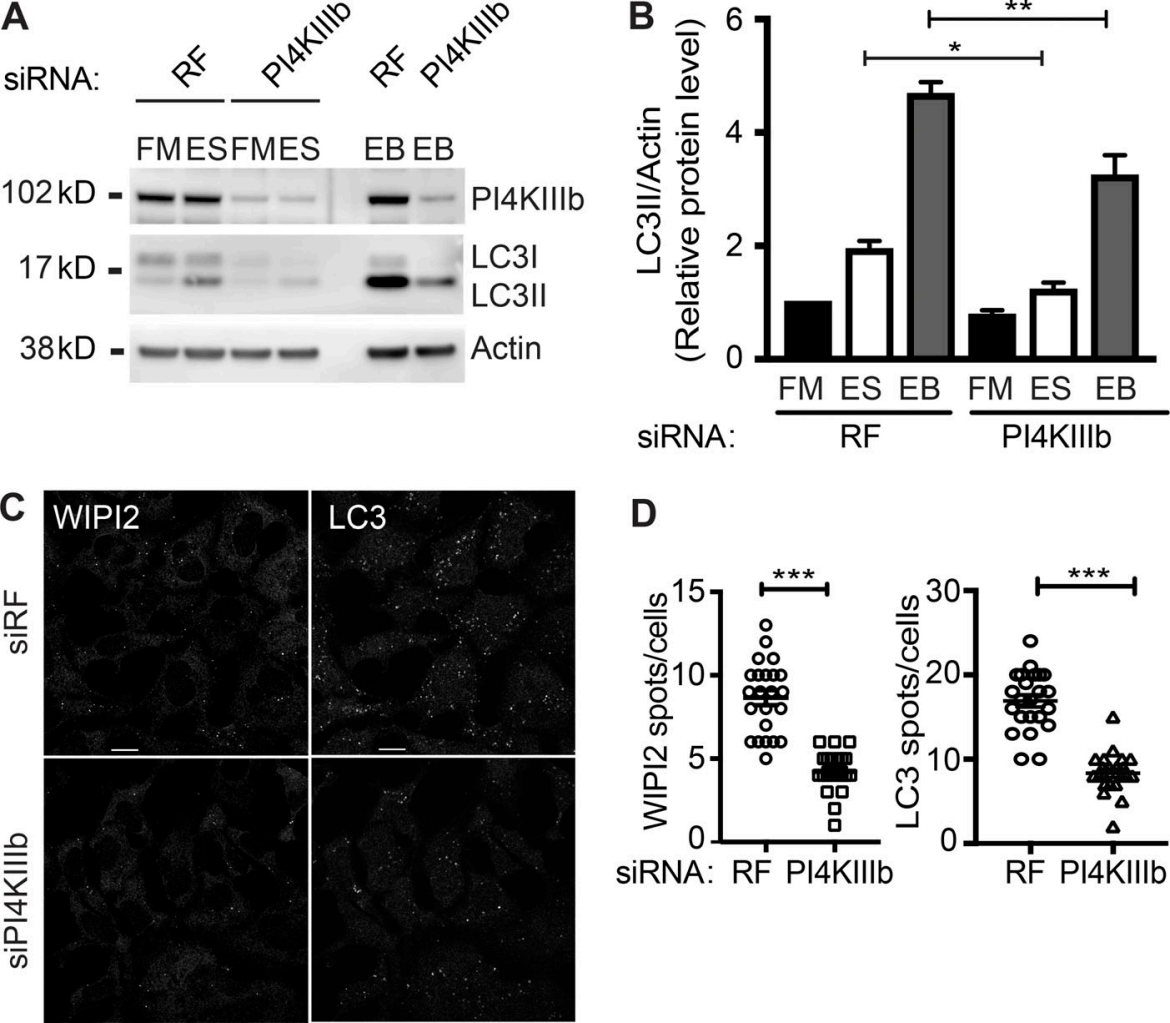
**PI4KIIIβ is a positive regulator of starvation-induced autophagy. (A)** HEK293A cells were treated with RF or PI4KIIIβ siRNA for 72 h and then incubated in FM or ES without or with Bafilomycin A1 (EB) for 2 h and immunoblotted for PI4KIIIβ, actin, and LC3B. **(B)** Quantification of A; statistical analysis using one-way ANOVA with Tukey’s multiple comparisons test, mean ± SEM, *n* = 7 experiments; *, P ≤ 0.05; **, P ≤ 0.01. **(C)** HEK293A cells were treated with RF or PI4KIIIβ siRNA for 72 h, incubated in ES for 2 h, fixed, and labeled using antibodies to WIPI2 or LC3. Scale bars, 10 µm. **(D)** WIPI2 and LC3B puncta in cells treated as in C were counted. Statistical analysis using two-tailed unpaired Student’s *t* test, mean ± SEM, *n* = 3 experiments, 100 cells per condition per independent experiment were quantified; ***, P ≤ 0.001.

To examine whether PI4KIIIβ could contribute to autophagosome initiation and formation, we assessed the impact of its depletion on LC3 lipidation. Depletion of PI4KIIIβ reduced LC3B-II in ES and ES plus Bafilomycin A (Fig. 8, A and B). To understand the role of PI4KIIIβ during autophagy, we examined the effect of its depletion on markers for the different stages of autophagosome formation. The number of WIPI2 and LC3B puncta were decreased in PI4KIIIβ-depleted cells, confirming a decrease in the rate of formation of phagophores and autophagosomes (Fig. 8, C and D). This shows that PI4KIIIβ acts at an early stage in the formation of autophagosomes, most likely at the initiation step.

### PI4KIIIβ acts at initiation sites containing ATG13

**Figure 9.**
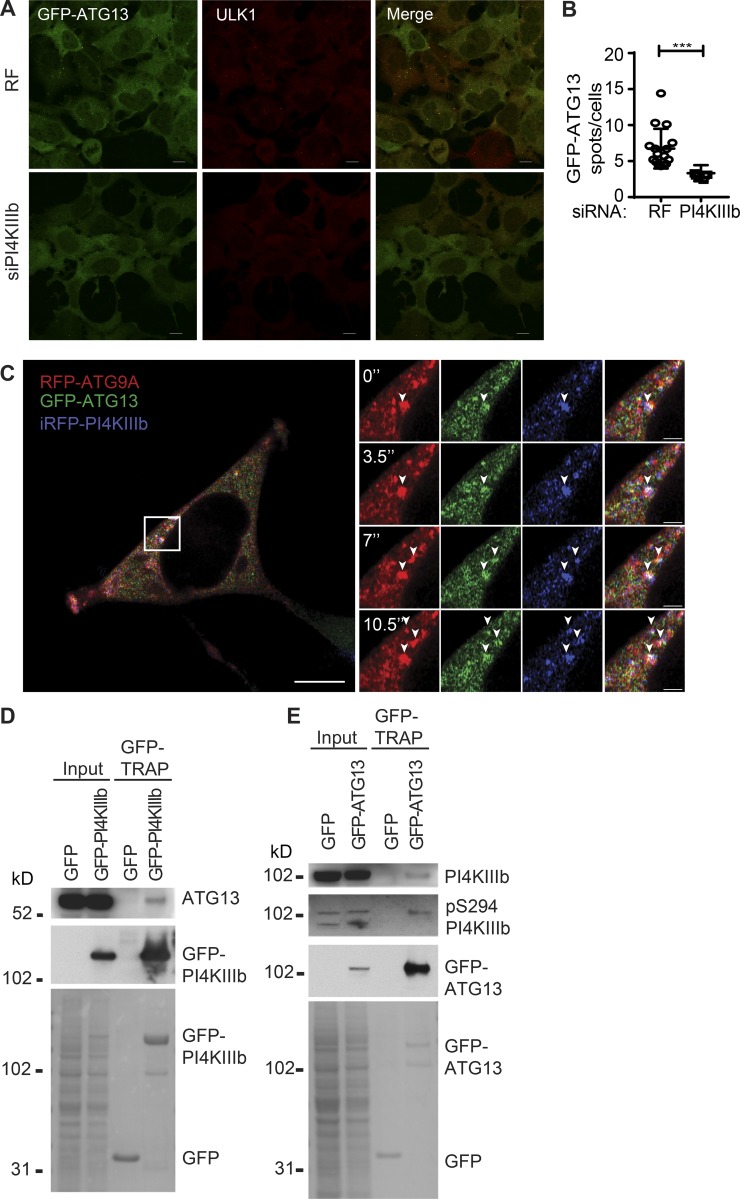
**PI4KIIIβ interacts with ATG13. (A)** HEK293A-GFP-ATG13 cells were treated with RF or PI4KIIIβ siRNA for 72 h and then incubated in ES for 2 h, fixed, and labeled using antibody to ULK1. Scale bars, 10 µm. **(B)** GFP-ATG13 puncta in cells treated as in A were counted. Statistical analysis using two-tailed unpaired Student’s *t* test, mean ± SEM, *n* = 3 experiments, 100 cells per condition per independent experiment were quantified; ***, P ≤ 0.001. **(C)** HEK293A stably expressing GFP-ATG13 and mRFP-ATG9A were transfected with iRFP-PI4KIIIβ, incubated in ES, and imaged live by Airyscan microscopy. Scale bars, 5 µm; inset, 1 µm. **(D)** GFP-TRAP pulldown of HEK293A cells transiently expressing GFP-PI4KIIIβ incubated in ES for 2 h followed by immunoblot for GFP and ATG13. **(E)** GFP-TRAP pulldown of HEK293A-GFP-ATG13 cells incubated in ES for 2 h followed by immunoblot for GFP, PI4KIIIβ, and pS294 PI4KIIIβ. In H and I, bottom panel is Ponceau S staining of input and GFP-TRAP.

PI4KIIIβ was found on GFP-ATG13–positive membranes (Fig. 6, F and G), which are the among the earliest autophagy-specific structures. ATG13 is a member of the ULK1/2 complex, which initiates autophagosome formation. As the PI4KIIIβ localizes more to GFP-ATG13 spots than LC3B spots (Fig. 6, C and D), we assessed the contribution of PI4KIIIβ to ATG13-positive phagophore formation. Depletion of PI4KIIIβ significantly reduced the number of GFP-ATG13 spots (Fig. 9, A and B).

As PI4KIIIβ is present on ATG9A vesicles, we asked if it trafficked like ATG9A. We performed live-cell imaging of cells stably expressing GFP-ATG13 and mRFP-ATG9A with iRFP-PI4KIIIβ transiently expressed under amino acid starvation (Fig. 9 C). As seen in Video 3, iRFP-PI4KIIIβ present on mRFP-ATG9 vesicles transiently interacted with GFP-ATG13–positive omegasomes. Furthermore, in detergent, GFP-PI4KIIIβ interacted with endogenous ATG13, and reciprocally GFP-ATG13 interacted with endogenous PI4KIIIβ and activated PI4KIIIβ pSer294 (Fig. 9, D and E).

Our results suggest that the ATG9A-positive membranes exiting from the Golgi complex to form the ATG9A compartment contain PI4KIIIβ. PI4KIIIβ is required for the formation of the ATG9A vesicle and is acting to promote the earliest stages of autophagosome formation.

### PI4KIIIβ acts at initiation sites containing ATG13 independently of PI3P production

**Figure 10.**
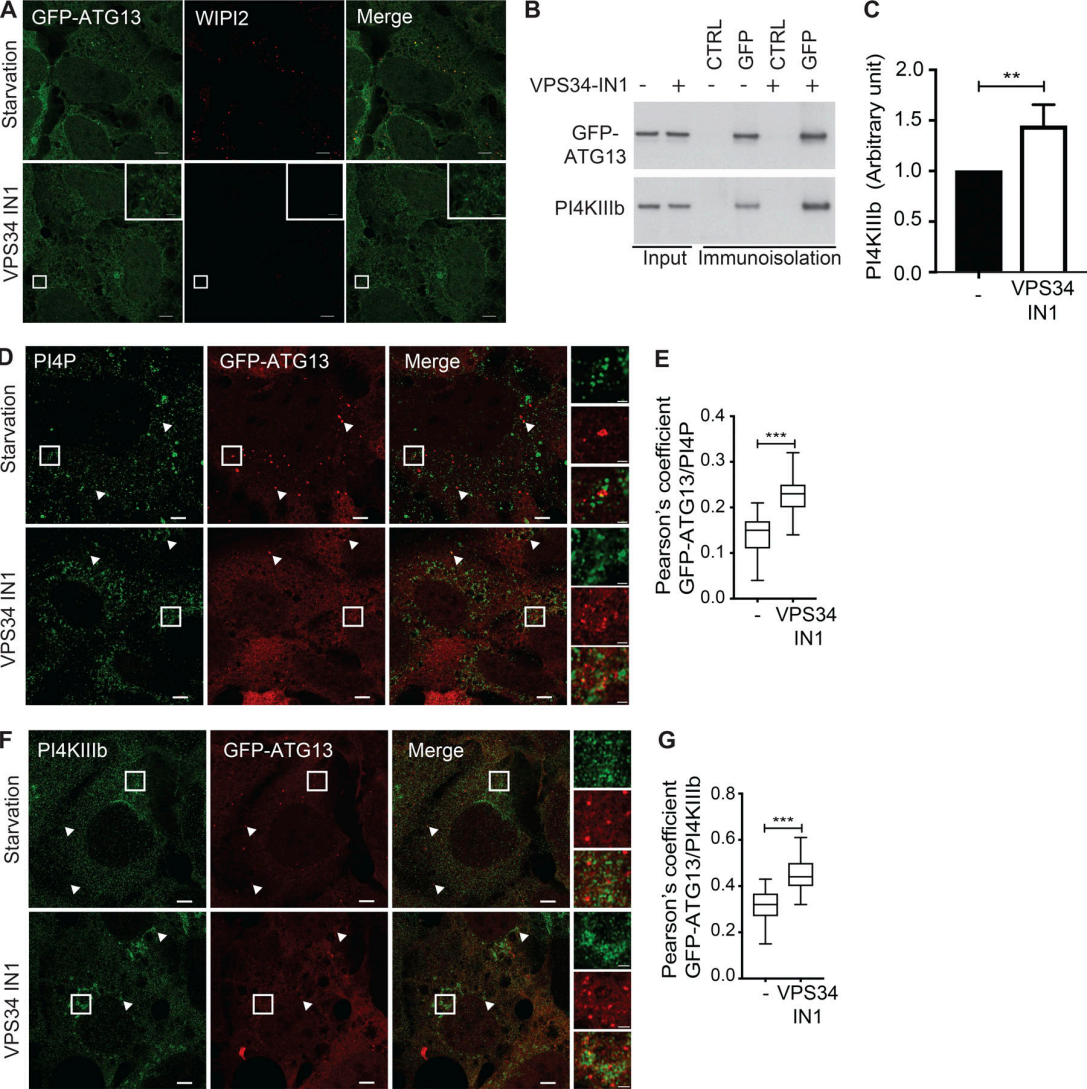
**PI4KIIIβ acts at initiation sites of autophagosomes in the absence of PI3P. (A)** HEK293A-GFP-ATG13 were incubated in ES with or without VPS34 IN1 for 1 h, fixed, and labeled for WIPI2. Airyscan imaging. Scale bars, 5 µm; inset, 1 µm. **(B)** HEK293A cells stably expressing GFP-ATG13 were incubated in ES with or without VPS34 IN1 for 1 h, and the GFP-positive compartment was immunoisolated before immunoblot for GFP and PI4KIIIβ. **(C)** Quantification of the relative amount of PI4KIIIβ after normalization to immunoisolated GFP-ATG13. Statistical analysis using two-tailed unpaired Student’s *t* test, mean ± SEM, *n* = 3 experiments; **, P ≤ 0.01. **(D)** HEK293A-GFP-ATG13 cells were incubated in ES with or without VPS34 IN1 for 1 h, fixed, and labeled using antibodies to PI4P. Airyscan imaging. Scale bars, 5 µm; inset, 1 µm. Arrowheads indicate colocalized structures. **(E)** Quantification of GFP-ATG13 and PI4P colocalization in D; statistical analysis using one-way ANOVA with Tukey’s multiple comparisons test, mean ± SEM, *n* = 3 experiments, Pearson’s coefficient of 30 cells per condition per independent experiment were quantified; ***, P ≤ 0.001. **(F)** HEK293A-GFP-ATG13 cells were incubated in ES with or without VPS34 IN1 for 1 h, fixed, and labeled using antibodies to PI4KIIIβ. Airyscan imaging. Scale bars, 5 µm; inset, 1 µm. Arrowheads indicate colocalized structures. **(G)** Quantification of GFP-ATG13 and PI4KIIIβ colocalization in F; statistical analysis using one-way ANOVA with Tukey’s multiple comparisons test, mean ± SEM, *n* = 3 experiments, Pearson’s coefficient of 30 cells per condition per independent experiment were quantified; ***, P ≤ 0.001.

To determine the role of PI4KIIIβ and PI4P in the early stages of autophagosome formation, we investigated the possibility that the PI3P-independent recruitment of ATG13 to initiation sites (Karanasios et al., 2016) was due to the presence of PI4P produced by PI4KIIIβ. The VPS34 inhibitor (VPS34-IN1; Bago et al., 2014), as expected, abolishes the formation of WIPI2 puncta but, as previously shown (Karanasios et al., 2016), not the formation of ATG13 puncta (Fig. 10 A). We immunoisolated GFP-ATG13–positive membranes after amino acid starvation in the presence of VPS34 IN1 and observed that GFP-ATG13–immunoisolated membranes contain PI4KIIIβ, which is increased with VPS34IN1 treatment (Fig. 10, B and C). We then examined the localization of PI4P after VPS34-IN1 treatment. Compared with amino acid starvation alone, addition of VPS34-IN1 increased colocalization between PI4P and GFP-ATG13 (Fig. 10, D and E). Furthermore, similar results were observed by localizing PI4KIIIβ and GFP-ATG13 (Fig. 10, F and G), supporting the notion that PI4KIIIβ is present and acting at the earliest stage of autophagosome formation, independently of PI3P synthesis. These results suggest that PI4P could be upstream of PI3P in initiation of autophagosomes.

## Discussion

Autophagy requires the formation of double-membrane autophagosomes, which can sequester cytoplasmic material and deliver it to lysosomes for degradation. Formation of the double membrane can be initiated by signaling pathways downstream of growth sensors such a mTORC1, but ultimately the unique mix of proteins and lipids needed for formation must be derived or delivered from the biosynthetic, secretory, or endocytic pathways.

While several ATG proteins have been directly implicated in these processes, there are few reports about the role of ATG9A in human disease, infection, and immunity. ATG9A is required to suppress *Salmonella* infection, as it drives the formation of the autophagosome surrounding invading *Salmonella* (Kageyama et al., 2011). In addition, the control of the immune response to double-stranded DNA by ATG9A is through trafficking of STING and assembly of a complex with TBK1. ATG9A negatively regulates STING–TBK1 complex assembly and translocation of STING from the Golgi complex into the compartment required for an innate immune response (Saitoh et al., 2010).

Here we addressed what is being delivered by ATG9A vesicles to the forming autophagosome. Under fed conditions, ATG9A mainly traffics though the Golgi and endosomes, but during amino acid starvation, its distribution is dramatically altered, and it resides in the vesicular-tubular ATG9 compartment (Orsi et al., 2012) or Atg9 reservoir in yeast (Mari et al., 2010). This vesicular ATG9A compartment interacts transiently with the ER and coalesces with ATG13 to nucleate autophagosome formation (Karanasios et al., 2016). We used SILAC-based immunoisolation to identify ATG9A-positive membranes that are enriched during starvation and identified a functional network on the ATG9A membranes comprised of BAR-domain membrane-shaping proteins, ARFIP1, ARFIP2, and BIF1 (SH3GLB1), and two PI4Ks that regulate initiation and maturation. We propose that ARFIP2 regulates ATG9A exit from the Golgi complex, thereby incorporating and delivering the PI4KIIIβ and PI4P to the autophagosome initiation site.

ARFIP2, and not ARFIP1, is required for initiation of autophagy. They share an overall homology of ∼80% (Kanoh et al., 1997), bind to ARL1 and ARF1, and are recruited to the Golgi complex (Cruz-Garcia et al., 2013). However, they have distinct functions; in particular, ARFIP2 binds RAC1 (Shin and Exton, 2001; Tarricone et al., 2001) and mediates cross-talk between RAC1 and ARF1 signaling pathways. Interestingly ARFIP2 regulates Htt aggregates (Peter et al., 2004), and AKT phosphorylation of ARFIP2 limits the accumulation of poly-Q Htt, thus acting as a neuroprotective mechanism (Rangone et al., 2005).

Overall, the function of ARFIPs is proposed to regulate cargo exit from the Golgi. ARFIP1 controls the exit of secretory granule components from the TGN (Gehart et al., 2012), while ARFIP2 takes enzymes such as the matrix metalloproteases destined for constitutive secretion into post-Golgi carriers and tubules (Man et al., 2011; Eiseler et al., 2016). We identified a role for ARFIP2 in autophagy and showed that the AH is required even in the presence of a functional BAR-domain. The AH of ARFIP2 is required for association with highly curved tubules (Ambroggio et al., 2013) and may help in specifying the location of tubule formation by targeting a subdomain of the Golgi complex where PI4P is enriched (Cruz-Garcia et al., 2013).

We propose that ARFIP2 regulates ATG9A exit into the ATG9 compartment. However, it is not known if ARFIP2 can mediate membrane scission, and this may be provided by BIF1 working with Dynamin 2 (Takahashi et al., 2016), a process which might also involve Sorting Nexin 18 (Søreng et al., 2018), so far described only for ATG9A vesicles emerging from Rab11-positive membranes.

On the Golgi complex, selection of cargo for secretion and vesicle egress requires recruitment of ARFIP2 by association with ARL1 and PKD2, at sites where ARF1 is in its GTP-bound state (Eiseler et al., 2016). PKD2 can phosphorylate PI4KIIIβ, and phosphorylation of PI4KIIIβ at Serine 294 activates PI4KIIIβ, increasing the lipid kinase activity (Hausser et al., 2005).

During amino acid starvation, ARFIP2 may initiate ATG9A vesicle formation and activation of binding partners of ATG9A, in particular PI4KIIIβ. Activation of PI4KIIIβ by PKD2 phosphorylation might drive the production of PI4P on the ATG9A-positive compartment, or at the autophagosome initiation site. Furthermore, as ATG9A translocation to initiation sites precedes ATG13 recruitment (Karanasios et al., 2016), and ATG13 binds PI4P (Karanasios et al., 2013), the delivery of the PI4KIIIβ to this site may produce PI4P locally by recruiting ATG13 and the ULK1 complex. A similar mechanism may function in yeast, where Atg9 binding to the HORMA domain of Atg13 may stabilize this interaction at the yeast phagophore (Suzuki et al., 2015).

Thus, we have revealed that an essential function of ATG9A in autophagy may be to deliver PI4P to all stages, including omegasomes, phagophores, and autophagosomes, participating in autophagy. ARFIP2 plays an essential role in this function: ATG9A in the Golgi complex is mobilized into highly dynamic vesicles in equilibrium with the ATG9 compartment, which can then deliver the phosphoinositide-metabolizing enzymes PI4KIIα and PI4KIIIβ.

## Materials and methods

### Cell lines and cell culture

HEK293A and their derivatives were grown in FM composed of DMEM supplemented with 10% FCS and 4 mM l-glutamine. The cells stably expressing HEK293/GFP-ATG13 and HEK298/GFP-DFCP1 were a gift from N. Ktistakis (Babraham Institute, Cambridge, UK; Axe et al., 2008; Karanasios et al., 2016) and maintained in the presence of G418 at 400 µg/ml. The HEK293/GFP-LC3B cells are as previously described (Chan et al., 2007). Cells stably expressing HEK293/GFP-ATG13/RFP-ATG9A were a gift from N. Ktistakis and maintained in the presence of G418 at 400 µg/ml and Zeocin at 400 µg/ml. Cells stably expressing HEK293/RFP-ATG9A were maintained in the presence of G418 at 400 µg/ml.

The ARFIP2 KO cell line was generated by CRISPR/Cas9-mediated genome engineering using the CRISPR design tool provided by the F. Zhang laboratory (Broad Institute, MIT, Boston, MA). A target sequence in the fifth exon of human ARFIP2 was selected (clone 1 sgRNA ARFIP2, forward 5′-TTA​TCA​GAA​CGA​TTT​GGT​CG-3′; reverse 5′-CGA​CCA​AAT​CGT​TCT​GAT​AA-3′; and clone 2 sgRNA ARFIP2, forward 5′-GGC​ATC​ACC​CAG​TGC​ATG​CT-3′; reverse 5′-AGC​ATG​CAC​TGG​GTG​ATG​CC-3′). The appropriate oligonucleotide was cloned into Bbs1 site of pSpCas9(BB)-2A-GFP plasmid obtained from the laboratory of F. Zhang (Addgene; 48138) according to the cloning protocol provided by the laboratory. Following single-cell sorting on GFP-positive cells, colonies were screened for ARFIP2 deficiency by immunoblot.

To induce autophagy, cells were washed three times with PBS and incubated in ES for 2 h. Where indicated, cells were treated with 100 nM bafilomycin A1 (Calbiochem; 196000) for 2 h or VPS34 IN1 (Cayman Chemical; 17392) for 1 h.

### Antibodies

The antibodies used in this work are as follows. Mouse antibodies: anti-WIPI2 (Polson et al., 2010), anti-GM130 (BD Biosciences; 610822), anti-PI4KΙΙα (Santa Cruz; sc-390026), anti-GFP (CRUK; 4E12), anti-HA (Covance; MMS-101R), anti-PI4P (Echelon Bioscience; Z-P004), GAPDH (Millipore; MAB374), and Syntaxin 12/13 (SYSY; 110131). Rabbit antibodies: anti-ATG9A (STO215 and STO219; Young et al., 2006), anti-LC3 (Abcam; ab48394), anti-GM130 (Abcam; ab52649), anti-ULK1 (for Western blotting, Santa Cruz; sc-33182; for immunofluorescence, Cell Signaling; 8054 D8H5), anti-GABARAP (Abgent; AP1821a), anti-Actin (Abcam; ab8227), anti-ARFIP2 (Invitrogen; 40-2400), anti-HA (Covance; PRB-101P), anti-PI4KIIIβ (Upstate; 06578), anti-pS294 PI4KIIIβ (Hausser et al., 2005), and anti-ATG13 (Chan et al., 2009). The following antibodies were used: hamster antibody: anti-ATG9A (Webber and Tooze, 2010); sheep antibody: anti-TGN46 (Serotec, AHP500G); goat antibody: anti-ARFIP1 (clone I-19; Santa Cruz; sc-19246).

Secondary antibodies for immunofluorescence (from Life Technologies unless otherwise specified) were anti-rabbit IgG Alexa Fluor 488, 555, and 647; anti-mouse IgG Alexa Fluor 488, 555, and 647; and anti-hamster Cy3 (Jackson ImmunoResearch). HRP-conjugated secondary antibodies used for Western blotting were from GE Healthcare.

### cDNA

GFP-ARFIP2 was a gift from K. Nakayama (Kyoto University, Kyoto, Japan; Man et al., 2011). GFP-PI4KIIIβ was a gift from T. Balla (National Institutes of Health/National Institute of Child Health and Human Development, Bethesda, MD). GFP-P4MX2 was a gift from G. Hammond (University of Pittsburgh, Pittsburgh, PA; Hammond et al., 2014). ARFIP2 cDNA constructs were subcloned into Gateway entry vectors to facilitate the transfer of cDNA into a variety of expression vectors. Expression clones were made as described in the Gateway cloning technology instruction manual (Invitrogen) using the gateway expression vectors pDestHA (Lamark et al., 2003) and pDest53 (Invitrogen; 12288015). pDestHA-ARFIP2 W99A point mutation was generated by using QuikChange Multi Site-Directed Mutagenesis Kit (Agilent Technologies; 200515). iRFP-PI4KIIIβ was generated by subcloning PI4KIIIβ into pLVX-iRFP. Primer used for ARFIP2 W99A was 5′-AAG​TTT​GAC​ATC​GTC​AAG​AAA​GCG​GGC​ATC​AAC​ACC​TAT​AAG​TGC-3′.

### RNAi and DNA transfection

Lipofectamine 2000 (Thermo Fisher Scientific; 11668019) was used for transient transfection of cells according to the manufacturer’s instructions. DNA plasmids were used at 1 µg/ml. For RNAi, cells were transfected with the relevant siRNA oligonucleotide using oligofectamine (Invitrogen; 12252011), followed by a second transfection using Lipofectamine 2000 the following day, both according to the manufacturer’s instructions. Cells were harvested 72 h after transfection. The final concentration of siRNA oligonucleotides was 100 nM. siRNAs used (Dharmacon): D-001220-01 (RF, CTRL), D-020256-01 (ARFIP1), D-012820-01 (ARFIP2-01), D-012820-02 (ARFIP2-02), D-014294-02 (ATG9A), and D-006777-02 (PI4KIIIβ).

### Western blotting

Cells were lysed in ice-cold TNTE buffer (20 mM Tris, pH 7.4, 150 mM NaCl, 5 mM EDTA, and 0.4% Triton X-100) containing EDTA-free Complete Protease Inhibitor cocktail (Sigma-Aldrich; 5056489001) and phosphatase inhibitor cocktail (Roche; 04906837001). Lysates were cleared by centrifugation for 15 min at 13,000 rpm, heated at 65°C for 15 min, and resolved on NuPAGE BIS-Tris 4–12% gels (Life Technologies) followed by transfer onto a PVDF membrane (Millipore). Following incubation with primary and secondary antibodies, the Western blots were developed by enhanced chemiluminescence (Immobilon Classico Western HRP substrate, WBLUC0500; Immobilon Crescendo Western HRP substrate, WBLUR0500; Millipore) on Hyperfilm MP (8 × 10 inches; Thermo Fisher Scientific; 10627265) or by chemiluminescence detection using Amersham Imager 680 (GE Healthcare). Densitometry was performed by using ImageJ software (National Institutes of Health).

### Immunoprecipitation

Cells were lysed using IP buffer (50 mM Tris, pH 7.4, 150 mM NaCl, 5 mM EDTA, and 1% Triton X-100) and 1× EDTA-free complete protease inhibitor and phosphatase inhibitor cocktail, and the clarified lysates used for immunoprecipitation were incubated with either rabbit anti-ATG9A (Webber and Tooze, 2010) or rabbit IgG CTRL (rabbit anti-HA) coupled to protein A Dynabeads (Invitrogen; 10002D) overnight at 4°C. Beads were washed five times with wash buffer (50 mM Tris, pH 7.4, 150 mM NaCl, 5 mM EDTA, and 0.1% Triton X-100) and eluted with 2× Laemmli sample buffer at 65°C for 15 min before resolving by SDS-PAGE and Western blotting. GFP-tagged proteins were immunoprecipitated using GFP-TRAP beads (Chromotek; GTA-20) using the IP buffer.

### Immunoisolation

For immunoisolation of ATG9A-positive membranes, HEK293 cells were treated with FM or ES for 2 h. Cells were then washed in ice-cold PBS and harvested by centrifugation at 200 *g* at 4°C. Pellets were resuspended using ice-cold isotonic buffer A (20 mM Hepes, pH 7.4, 250 mM sucrose, and 1 mM EDTA) supplemented with EDTA-free complete protease inhibitor cocktail and phosphatase inhibitor cocktail and incubated for 15 min on ice. The resuspended pellet was then passed through a 27G needle 15 to 20 times for homogenization before clarification by centrifugation at 3,000 *g* at 4°C. Supernatants were used for incubation overnight at 4°C with hamster anti-ATG9A or hamster IgM CTRL coupled with protein A Dynabeads (Invitrogen; 10002D). The ATG9A-positive membranes on the beads were washed three times with isotonic buffer supplemented with 150 mM NaCl. The ATG9A-positive membranes on the beads were then eluted by competition with ATG9A peptide (HPEPVPEEGSEDELPPQVHK). The eluted ATG9A-positive membranes were then pelleted by centrifugation at 47,000 rpm for 1 h at 4°C. The pellet membranes were resuspended in 2× Laemmli sample buffer before being resolved by SDS-PAGE and Western blotting. For confocal microscopy, mRFP-ATG9A–positive membranes were immunoisolated following the protocol described above and then fixed with 2% PFA after the washing step.

For immunoisolation of GFP-P4MX2–, GFP-ATG13–, GFP-DFCP1–, and GFP-LC3B–positive membranes, mouse anti-GFP antibody or mouse anti-FLAG M2 (CTRL) coupled to protein A Dynabeads was used. After the overnight incubation of the supernatants with the antibody coupled to protein A Dynabeads, GFP-positive membranes were washed with the isotonic buffer supplemented with 75–150 mM NaCl and eluted in 2× Laemmli sample buffer before being resolved by SDS-PAGE and Western blotting.

### Mass spectrometry and data analysis

For SILAC experiments, HEK293 cells were grown in DMEM supplemented with 10% dialyzed FCS (Invitrogen; S181D), and growth medium was supplemented with a combination of either 100 mg per liter of light (^14^N, ^12^C) or heavy (^15^N, ^13^C) lysine and arginine (CNLM-291-H and CNLM-539-H; CK Isotopes). ATG9-positive membranes were isolated from SILAC HEK293 cells either from nutrient-rich condition or amino acid starvation, using a hamster monoclonal antibody specific to the C-terminal domain of ATG9 conjugated to magnetic beads. Eluted proteins were subjected to SDS-PAGE until the running front had migrated 2–3 cm into the gel. Proteins were in-gel digested using trypsin, and peptides were analyzed using a Q Exactive mass spectrometer coupled to an Ultimate3000 HPLC equipped with an EASY-Spray source (Thermo Fisher Scientific). Raw data were processed using MaxQuant v1.3.0.5, with SILAC selected as the quantification algorithm. The proteingroup.txt output table was imported into Perseus software for further processing, statistical analysis, and visualization. Data were analyzed to screen for proteins enriched on ATG9A-positive membrane in nutrient-rich and amino acid starvation conditions and filtered as follows. Of all proteins identified, only those that were detected with a higher intensity in the specific ATG9A immunoisolation compared with IgM CTRL immunoisolation were considered as candidates. For each of these proteins, the log2 values of the normalized SILAC ratios were plotted against the log10 of the intensity to generate the scatterplot depicted in Figs. 1 B and 2 G. Relevant GO categories were projected onto the filtered log2 SILAC ratios for illustration purposes (Fig. 1 C). Raw mass spectrometry data and MaxQuant output files have been deposited to the ProteomeXchange Consortium (http://www.ebi.ac.uk/pride/archive/) via the PRIDE partner repository.

### Confocal microscopy

Cells were grown to subconfluence on coverslips and treated with FM or ES for 2 h. Cells were fixed by addition of 1 volume of prewarmed 4% PFA to the medium for 15 min at room temperature. Cells were then washed three times using PBS containing 50 mM NH_4_Cl. Cells were permeabilized with 20 µM digitonin (Merck Millipore; 300410), 0.2% Triton X-100 (ULK1 staining), or room temperature methanol (endogenous LC3 staining) for 5 min. Coverslips were blocked with 5% BSA (10735086001; Roche) for 1 h, incubated with primary antibody in 1% BSA for 1 h, washed with PBS, and incubated with secondary antibody in 1% BSA for 30 min, before final washing with PBS and MilliQ water. Cells were stained with Hoechst 33258 solution (23491-45-4; Sigma-Aldrich) and mounted with Mowiol 4-88 (475904; Calbiochem).

For PI4P staining, cells were permeabilized for 5 min with 20 µM digitonin in buffer A containing 20 mM Pipes, pH 6.8, 137 mM NaCl, and 2.7 mM KCl. Cells were washed three times in buffer A and then blocked for 45 min in buffer A containing 5% BSA. Anti-PI4P antibody was added in buffer A for 60 min at room temperature. After three washes with buffer A, secondary fluorophore-labeled antibodies were added in buffer A. Cells were postfixed for 5 min with 2% PFA, washed three times with PBS containing 50 mM NH_4_Cl and once with MilliQ water, and mounted.

For conventional confocal microscopy, a confocal laser microscope (upright LSM 710 confocal microscope system; Zeiss) with a 63× NA 1.46 plan-Apochromat objective was used. Superresolution microscopy was performed with Zeiss LSM 880, Airyscan-equipped confocal microscope with a 63× NA 1.4 plan-Apochromat objective. After acquisition, images were processed using an Airyscan processing tool on the ZEN software provided by Zeiss. Image channels were acquired sequentially using appropriate filter sets for DAPI, EGFP, mRFP, iRFP, and Alexa Fluor 555, 488, and 647 and combined in the corresponding microscope software. All imaging was performed at room temperature.

For conventional confocal microscopy, a confocal laser microscope (upright LSM 710 confocal microscope system) with a 63× oil-immersion lens was used. High-resolution confocal microscopy was performed with a Zeiss LSM 880, Airyscan-equipped confocal microscope with a 63× oil-immersion lens. After acquisition, images were processed using an Airyscan processing tool on the ZEN software provided by Zeiss.

The experiments were repeated as indicated in the figure legends, and representative images are shown. The level of colocalization was determined by acquiring confocal images from ∼30 cells per condition. Pearson’s coefficient was then determined using Fiji software. LC3, WIPI2, and ULK1 puncta formation was quantified by Imaris image analysis software. Line scans have been made by using Fiji software. Association between GFP-ATG13 spots with the different markers (PI4P, PI4KIIIβ, and WIPI2), GFP-LC3 spots with PI4P, and endogenous LC3 spots with GFP-PI4KIIIβ and with WIPI2 were analyzed with Fiji software based on the Manders’ overlap coefficient. Additionally, lines were drawn crossing GFP-ATG13, GFP-LC3B, or LC3 spots associated with the different markers, and line plots of the fluorescence intensity were used to confirm the association. In all experiments, images shown in individual panels were acquired using identical exposure times or scan settings and adjusted identically for brightness and contrast using Photoshop CS5 (Adobe).

### Live-cell imaging

Live-cell imaging was performed on HEK293A cells stably expressing GFP-ATG13 (HEK293A-GFP-ATG13) and mRFP-ATG9A cotransfected with iRFP-ARFIP2 or GFP-ATG13 and mRFP-ATG9A cotransfected with iRFP-PI4KIIIβ or on HEK293 cells stably expressing mRFP-ATG9A cotransfected with GFP-P4MX2 seeded on glass-bottom microwell dishes (MatTek Corp.; P35G-1.5-14-C). Cells were placed into ES at 37°C and 5% CO_2_ and immediately imaged using a Zeiss LSM 880, Airyscan-equipped confocal microscope with a 63× NA 1.4 plan-Apochromat objective. Images were processed using an Airyscan processing tool on the ZEN software provided by Zeiss.

### Statistical analysis

The statistical details of all experiments are reported in the figures, including statistical analysis performed, error bars, statistical significance, and exact *n* numbers. Statistics were performed using GraphPad Prism 6 software, as detailed in the figure legends.

### Online supplemental material

Fig. S1 provides evidence that in contrast to ARFIP2, ARFIP1 is not required for autophagy. Fig. S2 provides evidence that ARFIP2 is essential for autophagy. Fig. S3 shows the relationship between ATG9A and the PI4P pathway. Fig. S4 shows that the different stages of autophagosomes contain PI4P and PI4KIIIβ. Video 1 provides evidence that ATG9A vesicles containing ARFIP2 interact transiently with ATG13 vesicles. Video 2 shows that ATG9A vesicles contain PI4P. Video 3 shows that ATG13 vesicles interact with ATG9A vesicles containing PI4KIIIβ. Table S1 is a list of proteins associated with ATG9A-positive membranes under nutrient-rich and starvation conditions. Table S2 is a list of Golgi proteins associated with ATG9A-positive membranes. Table S3 is a list of proteins associated with, or depleted from, ATG9A-positive membrane upon ARFIP2 depletion.

## Supplementary Material

Supplemental Materials (PDF)

Tables S1-S3 (ZIP)

Video 1

Video 2

Video 3
