# Supplementary material for: ATG9A shapes the forming autophagosome through Arfaptin 2 and phosphatidylinositol 4-kinase IIIβ
Source: J Cell Biol. 2019 Mar 27;218(5):1634–52. doi: 10.1083/jcb.201901115 (PMC6504893; doi:10.1083/jcb.201901115)
Supplement: Supplemental Materials (PDF) [file JCB_201901115_sm.pdf]

JCB

Supplemental material

Judith et al., https://doi.org/10.1083/jcb.201901115

Provided online are three tables in Excel.

Table S1 lists proteins associated with ATG9A-positive membranes under nutrient-rich and starvation conditions. Raw data of proteins coimmunoisolated with ATG9A-positive membrane in nutrient-rich (FM) and starvation (ES) conditions shown in Fig. 1 B. Proteins are sorted according to their SILAC ratio and intensity.

Table S2 lists Golgi proteins associated with ATG9A-positive membranes. Raw data of proteins belonging to the Golgi GO category coimmunoisolated with ATG9A-positive membrane in nutrient-rich (FM) and starvation (ES) conditions (highlighted in red in Fig. S1 A). Proteins are sorted according to their SILAC ratio and intensity.

Table S3 lists proteins associated with, or depleted from, ATG9A-positive membrane in ARFIP2 CRISPR KO cells. Raw data of proteins coimmunoisolated with ATG9A-positive membrane in CTRL (ARFIP2 WT) and CrARFIP2 KO cells in starvation (ES) condition. Proteins are sorted according to their SILAC ratio and intensity.

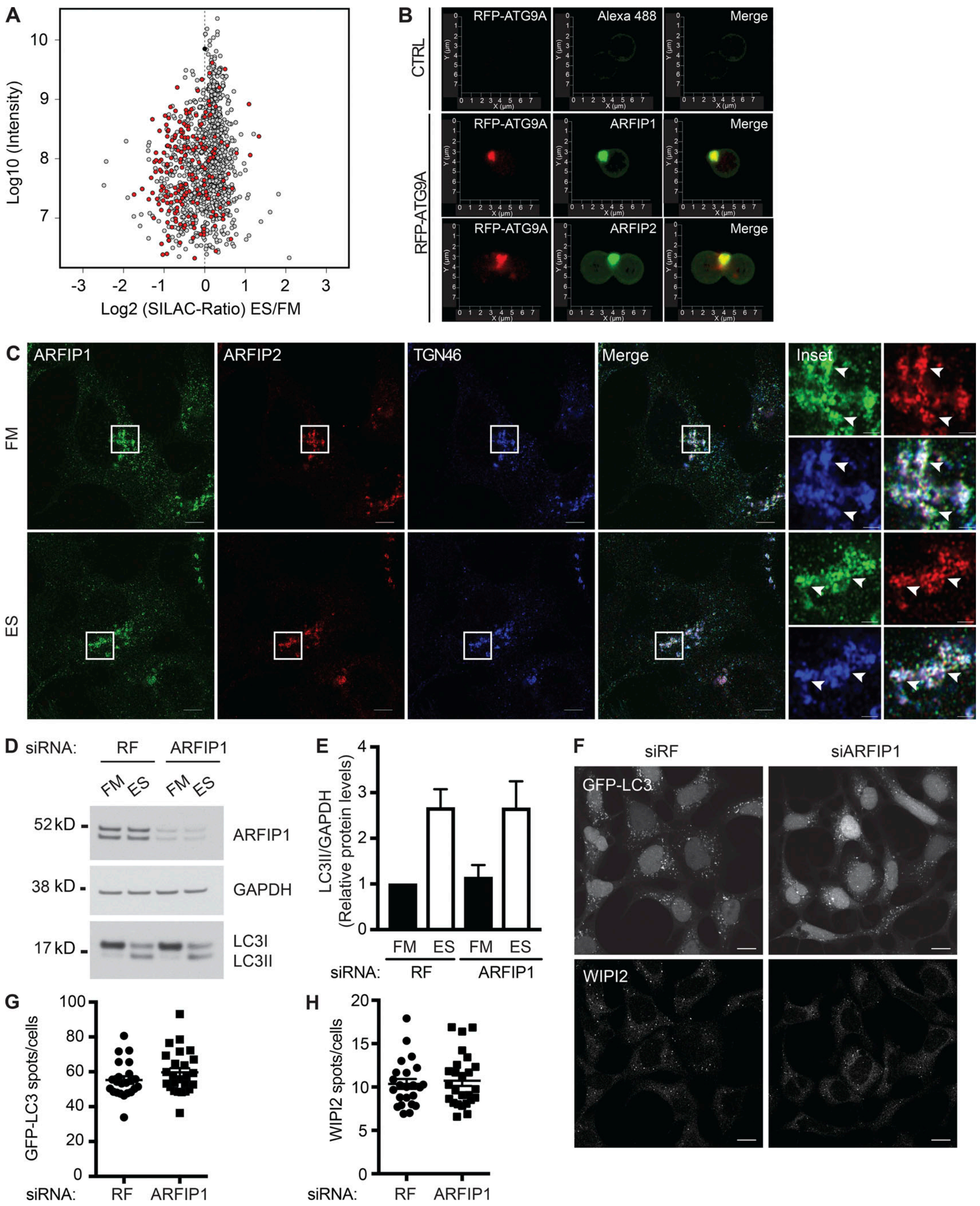

Figure S1. **ARFIP1 is not required for autophagy.** Related to Fig. 1. **(A)** Scatterplot of proteins associated with ATG9A-positive membranes from cells incubated in FM or under amino acid depletion (ES). The x axis displays the Log2 of the SILAC ratio (ES/FM), and the y axis displays the Log10 of the intensity of proteins found. Highlighted in black and red are ATG9A and proteins belonging to the Golgi apparatus GO category, respectively. See Table S2. **(B)** HEK293A cells stably expressing mRFP-ATG9A were incubated in ES for 2 h, and the ATG9A-positive compartment was immunoisolated with anti-ATG9A or IgM CTRL and fixed before immunostaining for ARFIP1 and ARFIP2. **(C)** HEK293A cells were incubated in FM or ES for amino acid starvation (ES) for 2 h, fixed, and labeled using antibodies to ARFIP1, ARFIP2, and TGN46. Airyscan imaging. Scale bars, 5 µm; inset, 1 µm. Arrowheads indicate colocalized structures. **(D)** HEK293A cells were treated with RF or Arfaptin 1 (ARFIP1) siRNA for 72 h and then incubated in FM or ES for 2 h and immunoblotted for ARFIP1, GAPDH, and LC3. **(E)** Quantification of D; mean ± SEM, *n* = 3 experiments. **(F)** HEK293A stably expressing GFP-LC3B cells were treated with RF or ARFIP1 siRNA for 72 h and then incubated in ES for 2 h, fixed, and labeled for WIPI2. Scale bars, 10 µm. **(G and H)** WIPI2 and GFP-LC3B puncta in F were counted. Mean ± SEM, *n* = 3 experiments, 100 cells per condition per independent experiment were quantified.

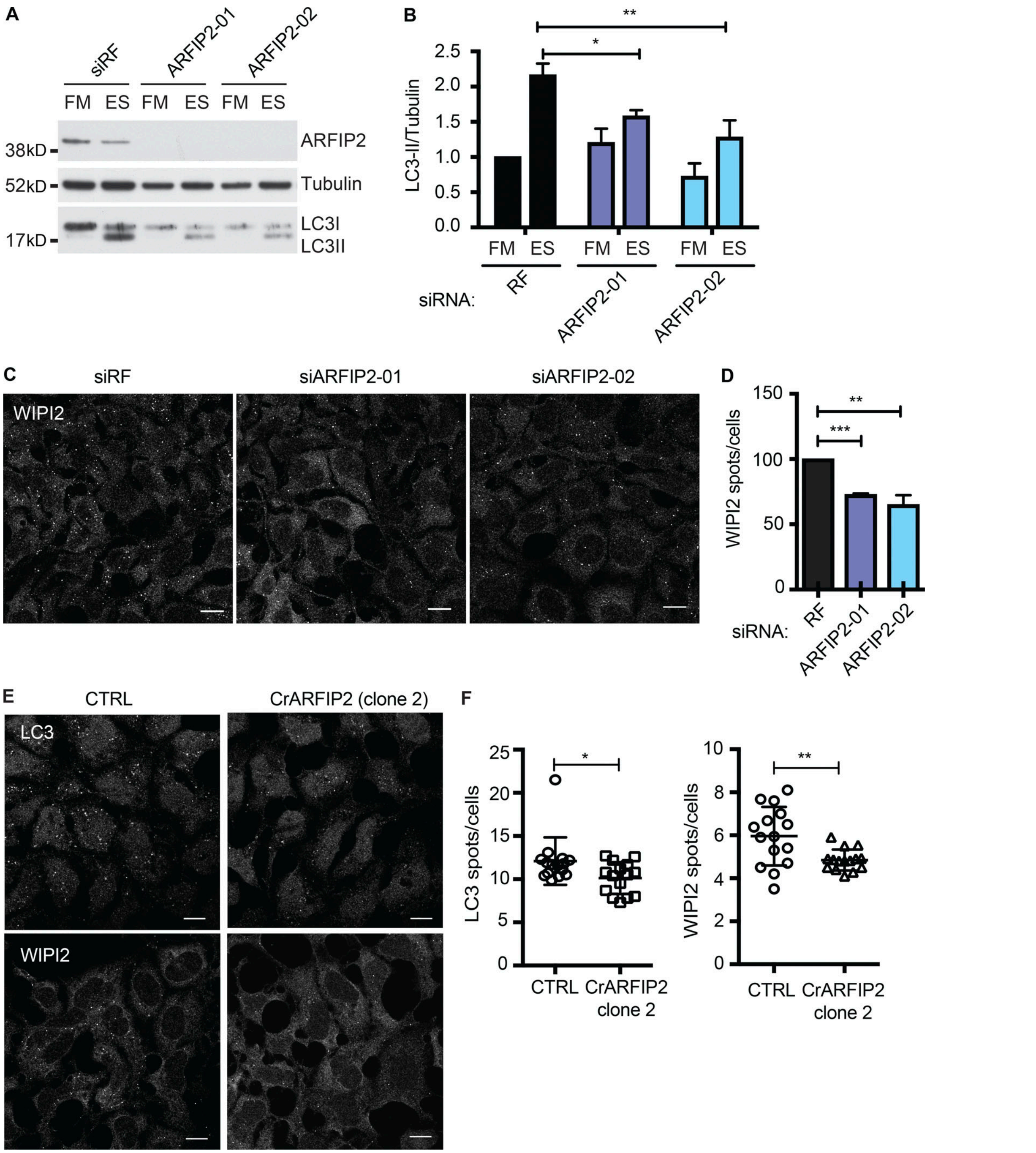

Figure S2. **ARFIP2 is needed for autophagy.** Related to Fig. 2. **(A)** HEK293A cells were treated with RF or Arfaptin 2 (ARFIP2) siRNA for 72 h and then incubated in FM or ES for 2 h and immunoblotted for ARFIP2, Tubulin, and LC3. **(B)** Quantification of A; mean ± SEM, *n* = 3 experiments. Statistical analysis using one-way ANOVA with Tukey's multiple comparisons test, mean ± SEM, *n* = 3 experiments; *, P ≤ 0.05; **, P ≤ 0.01. **(C)** HEK293A cells were treated with RF or ARFIP2 siRNA for 72 h and then incubated in ES for 2 h, fixed, and labeled for WIPI2. Scale bars, 10 µm. **(D)** WIPI2 puncta in C were counted. Statistical analysis using one-way ANOVA with Tukey's multiple comparisons test, mean ± SEM, *n* = 3 experiments, 100 cells per condition per independent experiment were quantified; **, P ≤ 0.01; ***, P ≤ 0.001. **(E)** HEK293A CTRL or CrARFIP2 KO (clone 2) cells were incubated in ES for 2h h, fixed, and labeled using antibodies to LC3B or WIPI2. Scale bars, 10 µm. **(F)** LC3B and WIPI2 puncta in A were counted. Statistical analysis using two-tailed unpaired Student's *t* test, mean ± SEM, *n* = 3 experiments, 100 cells per independent experiment; *, P ≤ 0.05; **, P ≤ 0.01.

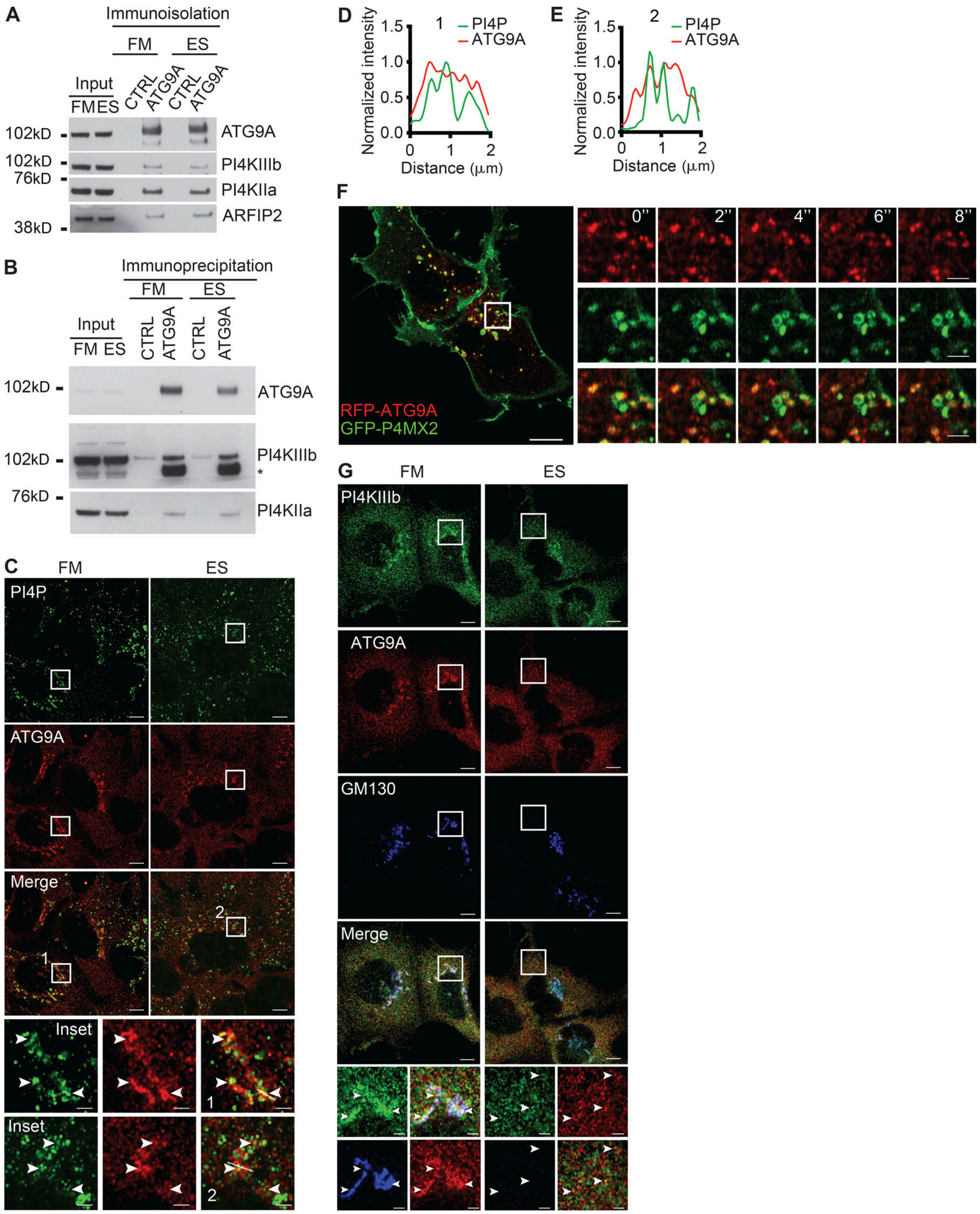

Figure S3. **ATG9A interacts with PI4KIIIβ and colocalizes with PI4P.** Related to Fig. 4. **(A)** HEK293A cells were incubated in FM or ES for 2 h, and the ATG9A-positive compartment was immunoisolated before immunoblot for ATG9A, ARFIP2, PI4KIIα, and PI4KIIIβ. **(B)** HEK293A cells were incubated in FM or ES for 2 h, lysed, and subjected to immunoprecipitation with rabbit anti-ATG9A or nonspecific rabbit IgG before immunoblot for ATG9A, PI4KIIα, and PI4KIIIβ. *, Nonspecific band. **(C)** HEK293A cells were incubated in FM or ES for 2 h, fixed, and labeled using antibodies to PI4P and ATG9A. Airyscan imaging. Scale bars, 5 µm; inset, 1 µm. Arrowheads indicate colocalized structures. **(D and E)** Line scans of FM (1) and line scans of ES (2) from C. **(F)** HEK293A stably expressing mRFP-ATG9A were transfected with GFP-P4MX2, incubated in ES, and imaged live by Airyscan microscopy. Scale bars, 5 µm; inset, 1 µm. **(G)** HEK293A cells incubated in FM or ES for 2 h, fixed, and labeled using antibodies to PI4K IIIβ, ATG9A, and GM130. Airyscan imaging. Scale bars, 5 µm; inset, 1 µm. Arrowheads indicate colocalized structures.

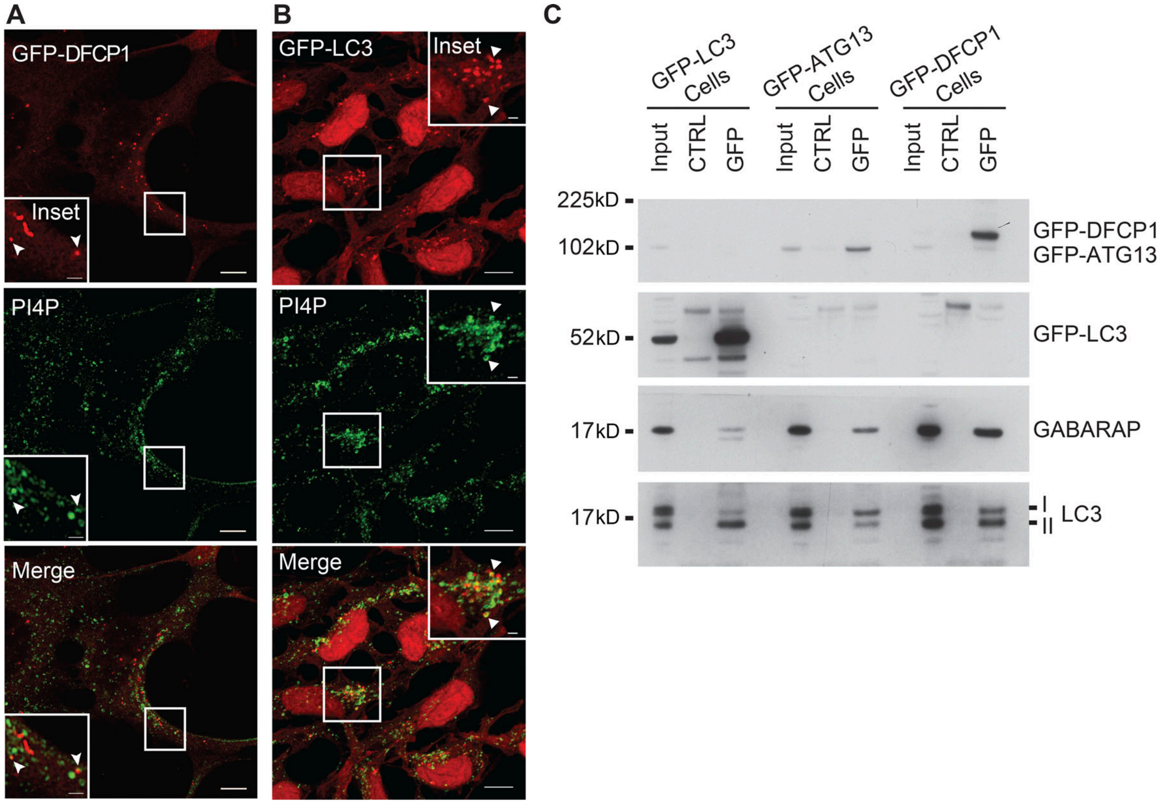

Figure S4. **Phagophores and autophagosomal membranes contain PI4P and PI4KIIIβ.** Related to Fig. 6. **(A)** HEK293A cells stably expressing GFP-DFCP1 were incubated in ES for 2 h, fixed, and labeled using antibodies to PI4P. Airyscan imaging. Scale bars, 5 µm; inset, 1 µm. **(B)** HEK293A cells stably expressing GFP-LC3B were incubated in ES for 2 h, fixed, and labeled using antibodies to PI4P. Airyscan imaging. Scale bars, 5 µm; inset, 1 µm. In A and B, arrowheads indicate colocalized structures. **(C)** HEK293A cells stably expressing GFP-LC3B, GFP-ATG13, and GFP-DFCP1 were incubated in ES for 2 h, and the GFP-positive compartments were immunoisolated before immunoblot for GFP, GABARAP, and LC3.

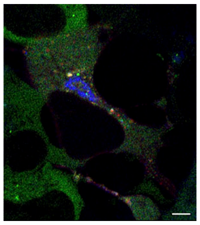

Video 1. **iRFP-ARFIP2 on mRFP-ATG9 vesicles transiently interacts with GFP-ATG13–positive membranes.** Live-cell imaging of a HEK293 cell stably expressing mRFP-ATG9A and GFP-ATG13 and transfected with iRFP-ARFIP2, incubated in EBSS (ES), and imaged live at 37°C with 10% CO_2 using a Zeiss LSM 880, Airyscan-equipped confocal microscope with a 63× NA 1.4 plan-Apochromat objective. Video spans 2 min played at 5 frames/s with 1 frame equal to 3 s.

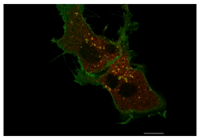

Video 2. **ATG9A-positive vesicles contain PI4P.** Live-cell imaging of a HEK293 cell stably expressing mRFP-ATG9A transfected with GFP-P4MX2, incubated in ES (ES), and imaged live at 37°C with 10% CO_2 using a Zeiss LSM 880, Airyscan-equipped confocal microscope with a 63× NA 1.4 plan-Apochromat objective. Video spans 1 min and 50 s played at 5 frames/s with 1 frame equal to 1 s.

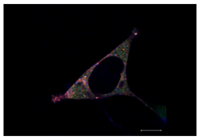

Video 3. **iRFP-PI4KIIIβ on mRFP-ATG9 vesicles transiently interacts with GFP-ATG13–positive membranes.** Live-cell imaging of a HEK293 cell stably expressing mRFP-ATG9A and GFP-ATG13 and transfected with iRFP-PI4KIIIβ, incubated in ES (ES), and imaged live at 37°C with 10% CO_2 using a Zeiss LSM 880, Airyscan-equipped confocal microscope with a 63× NA 1.4 plan-Apochromat objective. Video spans 2 min played at 5 frames/s with 1 frame equal to 3 s.
